# Post-translational control of beige fat biogenesis by PRDM16 stabilization

**DOI:** 10.1038/s41586-022-05067-4

**Published:** 2022-08-17

**Authors:** Qiang Wang, Huixia Li, Kazuki Tajima, Anthony R. P. Verkerke, Zachary H. Taxin, Zhishuai Hou, Joanne B. Cole, Fei Li, Jake Wong, Ichitaro Abe, Rachana N. Pradhan, Tadashi Yamamuro, Takeshi Yoneshiro, Joel N. Hirschhorn, Shingo Kajimura

**Affiliations:** 1 Division of Endocrinology, Diabetes and Metabolism, Beth Israel Deaconess Medical Center and Harvard Medical School, Boston, MA USA; 2 Department of Physiology and Pathophysiology, School of Basic Medical Sciences, Xi’an Jiaotong University Health Science Center, Xi’an, China; 3 Department of Endocrinology and Metabolism, National Hospital Organization, Yokohama Medical Center, Yokohama, Japan; 4 Programs in Metabolism and Medical & Population Genetics, Broad Institute of MIT and Harvard, Cambridge, MA USA; 5 Center for Genomic Medicine, Massachusetts General Hospital, Boston, MA USA; 6 Division of Endocrinology, Boston Children’s Hospital, Boston, MA USA; 7 Department of Pediatrics, Harvard Medical School, Boston, MA USA; 8 Department of Genetics, Harvard Medical School, Boston, MA USA; 9 Oncology Bioinformatics, Genentech, San Francisco, CA USA; 10 Division of Metabolic Medicine, Research Center for Advanced Science and Technology, The University of Tokyo, Tokyo, Japan; 11 Howard Hughes Medical Institute, Chevy Chase, MD USA

**Keywords:** Obesity, Metabolic syndrome, Ubiquitylation

## Abstract

Compelling evidence shows that brown and beige adipose tissue are protective against metabolic diseases^1,2^. PR domain-containing 16 (PRDM16) is a dominant activator of the biogenesis of beige adipocytes by forming a complex with transcriptional and epigenetic factors and is therefore an attractive target for improving metabolic health^3–8^. However, a lack of knowledge surrounding the regulation of PRDM16 protein expression hampered us from selectively targeting this transcriptional pathway. Here we identify CUL2–APPBP2 as the ubiquitin E3 ligase that determines PRDM16 protein stability by catalysing its polyubiquitination. Inhibition of CUL2–APPBP2 sufficiently extended the half-life of PRDM16 protein and promoted beige adipocyte biogenesis. By contrast, elevated CUL2–APPBP2 expression was found in aged adipose tissues and repressed adipocyte thermogenesis by degrading PRDM16 protein. Importantly, extended PRDM16 protein stability by adipocyte-specific deletion of CUL2–APPBP2 counteracted diet-induced obesity, glucose intolerance, insulin resistance and dyslipidaemia in mice. These results offer a cell-autonomous route to selectively activate the PRDM16 pathway in adipose tissues.

## Abstract (web summary)

The ubiquitin E3 ligase CUL2–APPBP2 determines PRDM16 protein stability by catalysing PRDM16 polyubiquitination in beige fat.

## Main

**Extended Data Fig. 1:**
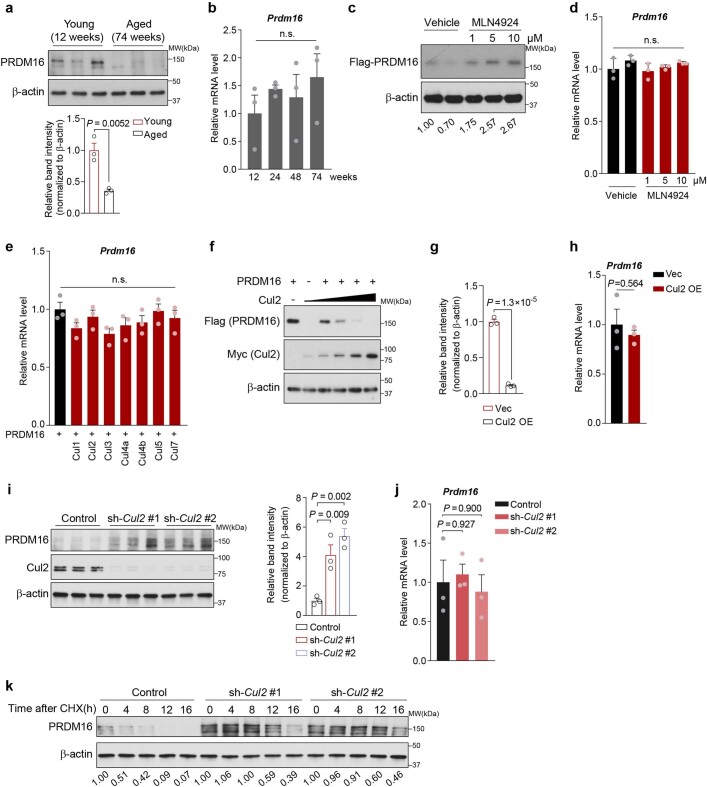
Regulation of PRDM16 protein and mRNA levels. **a**, Immunoblotting of indicated proteins in the inguinal WAT of mice at 12 and 74 weeks old. Lower panel: Quantification of PRDM16 protein levels and normalized to β-actin levels. **b**, Relative mRNA levels of *Prdm16* in the inguinal WAT of mice at indicated age. **c**, **d**, Immunoblotting of indicated proteins (**c**) and relative mRNA levels of *Prdm16* (**d**) in inguinal adipocytes expressing Flag-tagged PRDM16 in the presence or absence of MLN4924. **e**, Relative mRNA levels of *Prdm16* in HEK293T cells expressing indicated constructs. **f**, Immunoblotting of Flag-tagged PRDM16 protein in HEK293T cells expressing indicated constructs. **g**, Quantification of PRDM16 protein levels in Fig. 1b by Image J software and normalized to β-actin levels. **h**, Relative mRNA levels of *Prdm16* in inguinal adipocytes expressing Flag-tagged Cul2 or an empty vector. **i**, Left: Immunoblotting of endogenous PRDM16 and Cul2 in inguinal adipocytes expressing a scrambled control shRNA (Control) or two independent shRNAs targeting *Cul2* (#1, #2). Right: Quantification of PRDM16 protein levels by Image J software and normalized to β-actin levels. **j**, Relative mRNA levels of *Prdm16* in inguinal adipocytes expressing a scrambled control shRNA (Control) or two independent shRNAs targeting *Cul2*. **k**, Changes in endogenous PRDM16 protein stability in inguinal adipocytes expressing a scrambled control shRNA (Control) or shRNA targeting *Cul2*. PRDM16 protein levels were quantified by Image J software and normalized to β-actin levels. Representative results in **a**, **c**, **f**, **i**, **k** from two independent experiments. Gel source data are presented in Supplementary Fig. 1. **a**, **b**, **d**, **e**, **g**-**j**, *n* = 3 per group, biologically independent samples. Data are mean ± s.e.m.; two-sided *P* values by one-way ANOVA followed by Dunnett’s test (**b**, **d**, **e**, **i**, **j**) or unpaired Student’s *t*-test (**a**, **g**, **h**). n.s., not significant. Source data

Historically, targeting a transcriptional factor constitutes a major challenge unless specific ligands exist. Identifying the protein degradation machinery—that is, ubiquitin E3 ligases—offers substantial progress because it provides an alternative route to manipulate a specific transcriptional pathway of interest. Such successful examples include the characterization of mouse double minute 2 homologue (MDM2) for the p53 tumour suppressor protein^9^; Kelch-like ECH-associated protein 1 (KEAP1) for NF-E2 p45-related factor (NRF2)^10^; and Von Hippel–Lindau tumour suppressor (VHL) for hypoxia-inducible factor 1α (HIF-1α)^11,12^. Notably, several observations indicate that PRDM16 is dynamically regulated at the post-translational level. For example, PRDM16 protein expression was reduced in ageing with no change in its mRNA expression (Extended Data Fig. 1a,b). By contrast, overexpression of euchromatic histone-lysine *N*-methyltransferase 1 (EHMT1) or chronic treatment with synthetic ligands of peroxisome proliferator-activated receptor-γ (PPARγ) extends the half-life of PRDM16 protein and enhances adipose tissue thermogenesis^13,14^. However, the underlying mechanism responsible for PRDM16 protein degradation remains unclear.

A hint arose from our observation that PRDM16 protein levels, but not mRNA levels, were highly increased by MLN4924 (pevonedistat), a small-molecule inhibitor of protein neddylation (a NEDD8-activating enzyme inhibitor) (Extended Data Fig. 1c,d). Note that neddylation (that is, adding NEDD8 to target proteins such as cullin–RING E3 ubiquitin ligases) stimulates ubiquitin ligase activity^15,16^. Accordingly, we set out to search for the ubiquitin E3 ligase complex for PRDM16 protein.

## CUL2 reduces the stability of PRDM16 protein

**Fig. 1:**
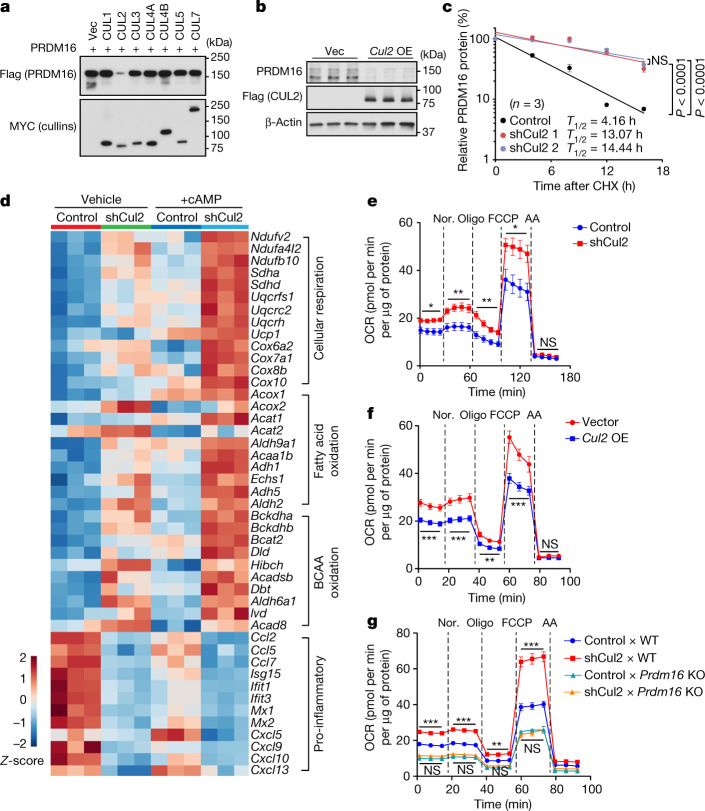
CUL2 controls PRDM16 protein stability and beige fat biogenesis. **a**, Immunoblotting of Flag-tagged PRDM16 protein in HEK293T cells co-expressing Myc-tagged cullin proteins or an empty vector (Vec). **b**, Immunoblotting of endogenous PRDM16 protein in inguinal adipocytes overexpressing (OE) Flag-tagged CUL2 or Vec. β-Actin was used as the loading control. **c**, Changes in endogenous PRDM16 stability in inguinal adipocytes expressing a scrambled control shRNA (control) or shRNAs targeting *Cul2* (1 and 2). Immunoblotting data are provided in Extended Data Fig. 1k. *n* = 3 per group. CHX, cycloheximide. **d**, Heat map of the RNA-seq transcriptome in differentiated inguinal adipocytes expressing a scrambled control (control) or shRNA targeting *Cul2* in the presence or absence of forskolin (+cAMP). *n* = 3 per group. All of the listed genes are significantly different (false-discovery rate (FDR) < 0.05) by edgeR. **e**, The OCR in differentiated inguinal adipocytes expressing a scrambled control shRNA or shCul2. OCR values were normalized by total protein (μg). *n* = 7 (control) and *n* = 6 (*Cul2* knockdown). AA, antimycin A; Nor., noradrenaline. **f**, The OCR in inguinal adipocytes expressing an empty vector or *Cul2* normalized by total protein (μg). *n* = 9 for both groups. **g**, The OCR in inguinal adipocytes expressing a scrambled control shRNA or shCul2 from WT and *Prdm16-*KO mice. *n* = 8 (control × WT), *n* = 7 (shCul2 × WT), *n* = 9 (control × *Prdm16* KO) and *n* = 9 (shCul2 × *Prdm16* KO). For **a** and **b**, representative results are shown from two independent experiments. Gel source data are presented in Supplementary Fig. 1. For **b**–**g**, data are from biologically independent samples. For **c** and **e**–**g**, data are mean ± s.e.m. Two-sided *P* values were calculated using two-way analysis of variance (ANOVA) (**c**) or two-way repeated-measures ANOVA (**e** and **f**) followed by Tukey test (**g**). *P < 0.05, **P < 0.01, ***P < 0.001; NS, not significant. Exact *P* values are shown in Supplementary Table 2. Source data

We first examined which cullin–RING family member is responsible for the effect of MLN4924 on PRDM16 protein accumulation. To this end, we ectopically expressed Flag-tagged PRDM16 together with each cullin–RING member (CUL1–7) and found that co-expression of CUL2, but none of the other family members, reduced PRDM16 protein expression without affecting *Prdm16* mRNA levels (Fig. 1a and Extended Data Fig. 1e,f). CUL2 overexpression in adipocytes also decreased endogenous PRDM16 protein expression without affecting its mRNA levels (Fig. 1b and Extended Data Fig. 1g,h). By contrast, depletion of *Cul2* by two independent shRNAs in inguinal white adipose tissue (IngWAT)-derived preadipocytes led to a concomitant upregulation of endogenous PRDM16 protein with no change in its mRNA expression (Extended Data Fig. 1i,j). Furthermore, cycloheximide chase assays showed that *Cul2* depletion significantly extended the half-life of PRDM16 protein from 4.2 h to 13.1–14.4 h (Fig. 1c and Extended Data Fig. 1k), which corroborated our previous research^13,14^.

**Extended Data Fig. 2:**
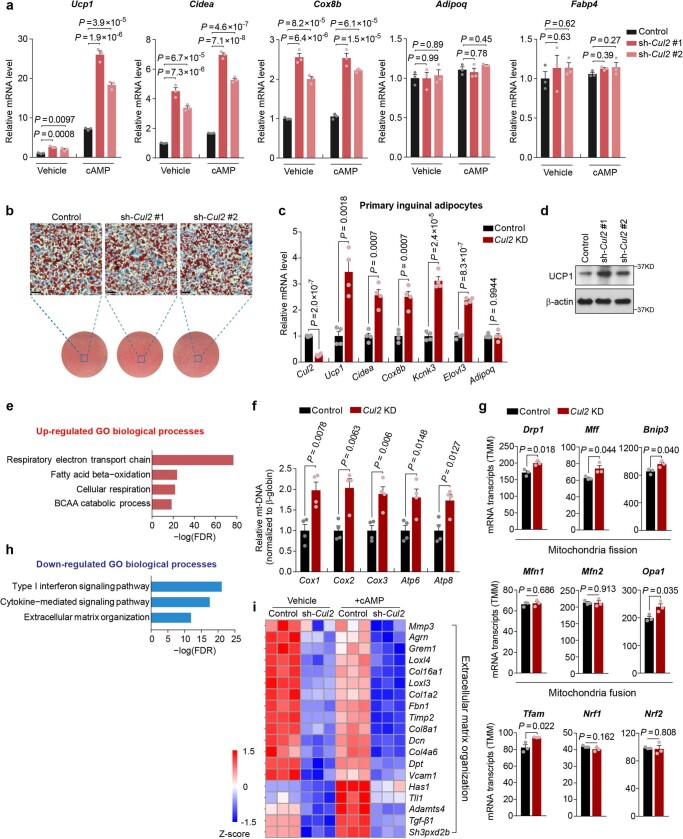
Depletion of *Cul2* promotes thermogenesis in white adipocytes. **a**, Relative mRNA levels of indicated genes in inguinal adipocytes expressing a scrambled control shRNA (Control) or shRNAs targeting *Cul2* with or without forskolin. *n* = 3 per group. **b**, Oil-Red-O staining of inguinal adipocytes stably expressing a scrambled control shRNA (Control) or shRNAs targeting *Cul2*. Scale bar, 100 μm. **c**, Relative mRNA levels of indicated genes in control and *Cul2* KD primary inguinal adipocytes. *n* = 4 per group. **d**, Immunoblotting of UCP1 and β-actin in inguinal adipocytes expressing a scrambled control shRNA (Control) or shRNAs targeting *Cul2*. **e**, Gene ontology (GO) analysis of RNA-seq data in Fig. 1d showing upregulated biological processes in *Cul2*-depleted inguinal adipocytes relative to control adipocytes. **f**, Indicated mitochondrial DNA transcripts in control and *Cul2* KD inguinal adipocytes. *n* = 4 per group. **g**, Expression levels (Trimmed Mean of M-values, TMM) of indicated genes in the differentiated inguinal adipocytes expressing a scrambled control (Control) or shRNA targeting *Cul2* (Cul2 KD). *n* = 3 per group. **h**, GO analysis of RNA-seq data in Fig. 1d showing downregulated biological processes in *Cul2*-depleted inguinal adipocytes relative to control adipocytes. **i**, Heat-map of transcriptome showing the changes in mRNA levels of indicated genes involving in the extracellular matrix organization in inguinal adipocytes expressing scrambled control (Control) or shRNA targeting *Cul2* (sh-*Cul2* #1) with or without forskolin (+ cAMP). *n* = 3 per group. All the listed genes are significantly different (FDR < 0.01) by edgeR. Representative results in **b** and **d** from two independent experiments. Gel source data are presented in Supplementary Fig. 1. **a**, **c**, **f**, **g**, **i**, biologically independent samples. Data are mean ± s.e.m.; two-sided *P* values by one-way ANOVA followed by Dunnett’s test (**a**) or unpaired Student’s *t*-test (**c**, **f**, **g**). Source data

**Extended Data Fig. 3:**
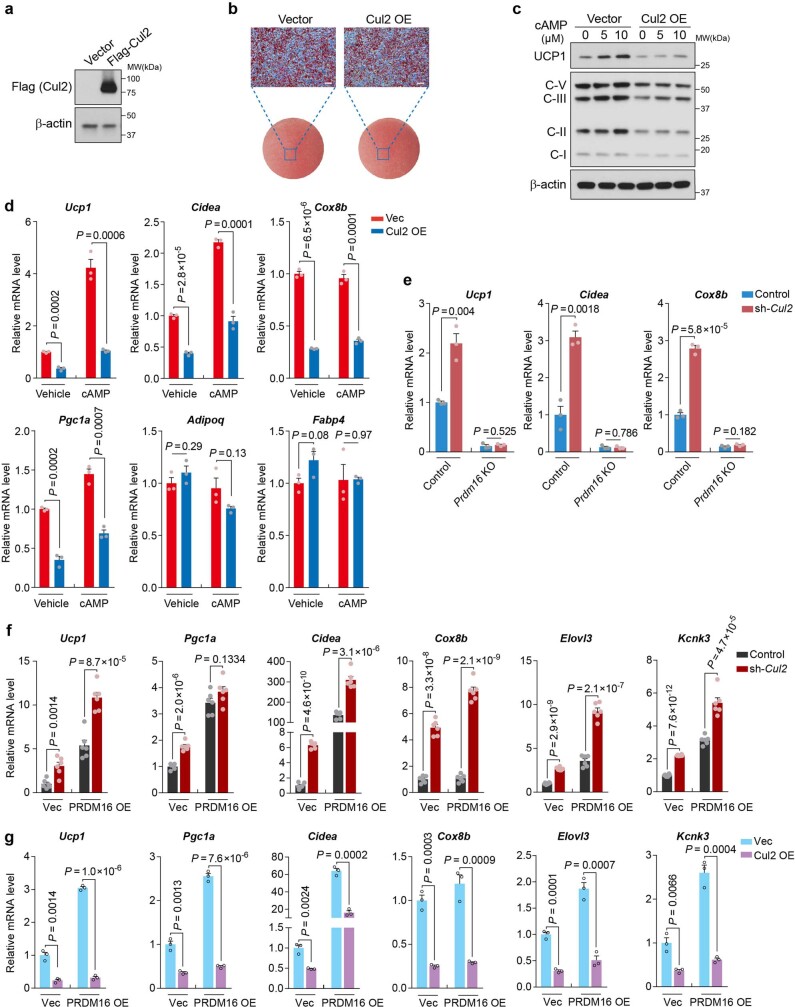
*Cul2* overexpression inhibits fat thermogenesis without affecting adipogenesis. **a**, Immunoblotting of Cul2 (Flag) proteins in inguinal adipocytes expressing an empty vector or Flag-tagged Cul2 (Cul2 OE). β-actin was used as a loading control. **b**, Oil-Red-O staining of inguinal adipocytes stably expressing an empty vector or Flag-tagged Cul2 (Cul2 OE). Scale bar, 50 μm. **c**, Immunoblotting for UCP1 and indicated mitochondrial proteins in inguinal adipocytes stably expressing an empty vector or Flag-tagged Cul2 (Cul2 OE). Differentiated inguinal adipocytes were treated with or without 5 μM or 10 μM forskolin (cAMP) for 8 h. β-actin was used as a loading control. **d**, Relative mRNA levels of indicated genes in inguinal adipocytes stably expressing an empty vector or Flag-tagged Cul2 in the presence or absence of forskolin (cAMP). *n* = 3 per group. **e**, Relative mRNA levels of indicated genes in control and *Prdm16* KO inguinal adipocytes expressing a scrambled control shRNA or shRNA targeting *Cul2*. *n* = 3 per group. **f**, Relative mRNA levels of indicated genes in differentiated inguinal adipocytes that expressed *Prdm16* (PRDM16 OE) or a control vector and a scrambled control shRNA or shRNA targeting *Cul2*. *n* = 6 per group. **g**, Relative mRNA levels of indicated genes in differentiated inguinal WAT-derived adipocytes expressing *Prdm16* (PRDM16 OE) or a control vector and/or Flag-tagged Cul2 (Cul2 OE). *n* = 3 per group. Representative results in **a**–**c** from two independent experiments. Gel source data are presented in Supplementary Fig. 1. **d**–**g**, biologically independent samples. Data are mean ± s.e.m.; two-sided *P* values by unpaired Student’s *t*-test (**d**–**g**). Source data

Stabilization of the PRDM16 protein by *Cul2* depletion substantially affected downstream metabolic cascades in primary and immortalized adipocytes without affecting adipogenesis (Fig. 1d and Extended Data Fig. 2a–d). For example, *Cul2* depletion significantly increased the expression of numerous PRDM16-target genes (for example, *Ucp1*), particularly when adipocytes were treated with forskolin. RNA-sequencing (RNA-seq) followed by biological pathway analysis showed that vital processes involving brown/beige fat function—including mitochondrial respiration, fatty acid oxidation and BCAA degradation—were upregulated by *Cul2* depletion (Fig. 1d and Extended Data Fig. 2e–g). By contrast, *Cul2* depletion significantly repressed pro-inflammatory pathways (cytokine signalling, type I interferon signalling) and pro-fibrotic pathways (extracellular matrix organization) (Fig. 1d and Extended Data Fig. 2h,i). These results are consistent with previous studies showing that PRDM16 activates the brown/beige-fat-selective gene program and mitochondrial BCAA and fatty acid oxidation, while repressing adipose tissue inflammation and fibrosis^3–6,17–20^. Importantly, *Cul2* depletion in white adipocytes significantly increased total and uncoupled cellular respiration as measured by oxygen-consumption rate (OCR) (Fig. 1e). In turn, overexpression of CUL2 in adipocytes reduced the expression of UCP1 and mitochondrial proteins and the mRNA levels of brown/beige-fat-selective genes without blocking adipogenesis, which was accompanied by decreased cellular respiration (Fig. 1f and Extended Data Fig. 3a–d).

CUL2 functions as a scaffold protein by interacting with several substrate receptors, including VHL^21,22^. We therefore investigated the degree to which CUL2 is responsible for controlling adipocyte thermogenesis through the regulation of PRDM16. To this end, we first depleted *Cul2* in inguinal WAT-derived adipocytes from wild-type (WT) or *Prdm16-*KO mice. We found that *Cul2* depletion significantly increased cellular OCR and the expression levels of *Ucp1, Cidea* and *Cox8b* in WT adipocytes, whereas this effect was blunted in *Prdm16-*KO cells (Fig. 1g and Extended Data Fig. 3e). Second, we depleted *Cul2* in inguinal WAT-derived adipocytes overexpressing PRDM16 or a control vector. Although PRDM16 overexpression potently activated the transcription of thermogenesis genes, depletion of *Cul2* further enhanced the stimulatory action of PRDM16 on these genes (Extended Data Fig. 3f). Third, we overexpressed *Cul2* in PRDM16-overexpressing adipocytes and found that CUL2 potently blunted the stimulatory effect of PRDM16 on the brown/beige-fat genes (Extended Data Fig. 3g).

## CUL2 loss in fat improves metabolic health

**Fig. 2:**
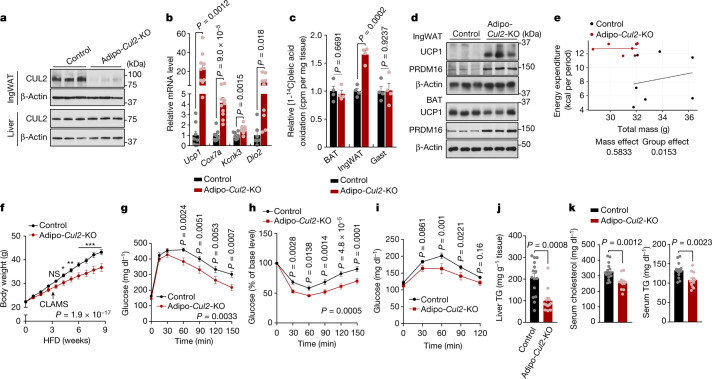
Fat-specific loss of *Cul2* counteracts diet-induced obesity, insulin resistance and dyslipidaemia. **a**, Immunoblot analysis of CUL2 protein in the inguinal WAT (IngWAT) and liver of Adipo-*Cul2*-KO and littermate control mice. *n* = 3 per group. **b**, Relative mRNA levels of the indicated genes in the inguinal WAT of Adipo-*Cul2*-KO mice (*n* = 10) and littermate controls (*n* = 8) on a regular chow diet at 30 °C. **c**, Oleic acid oxidation normalized to tissue mass (mg) in the interscapular BAT, inguinal WAT and gastrocnemius (Gast) muscle of mice on a regular chow diet. *n* = 4 per group. cpm, counts per minute. **d**, Immunoblot analysis of UCP1 and PRDM16 in the inguinal WAT and BAT of mice on an HFD. *n* = 3 per group. **e**, Regression-based analysis of energy expenditure against body mass. *n* = 7 per group. Data were analysed using CaIR-ANCOVA with energy expenditure as a dependent variable and body mass as a covariate. *P* values are shown at the bottom. **f**, Body weight of Adipo-*Cul2*-KO mice (*n* = 12) and littermate controls (*n* = 15) on an HFD at 22 °C. CLAMS, comprehensive laboratory animal monitoring system. **g**, Glucose-tolerance test of mice at 9 weeks of HFD. **h**, Insulin-tolerance test of mice at 10 weeks of HFD. **i**, Pyruvate-tolerance test of mice at 11 weeks of HFD. **j**, Triglyceride (TG) content in the liver of mice at 14 weeks of HFD. **k**, Serum cholesterol and triglyceride levels of the mice in **j**. For **a** and **d**, representative results from two independent experiments are shown. Gel source data are presented in Supplementary Fig. 1. For **a**–**k**, data are from biologically independent samples. For **b**, **c** and **f**–**k**, data are mean ± s.e.m. Two-sided *P* values were calculated using unpaired Student’s *t*-tests (**b**, **c**, **j** and **k**) or two-way repeated-measures ANOVA (**f**–**h**) followed by Fisher’s least significant difference test (**f**–**i**). *P < 0.05, **P < 0.01, ***P < 0.001. Exact *P* values are shown in Supplementary Table 2. Source data

**Extended Data Fig. 4:**
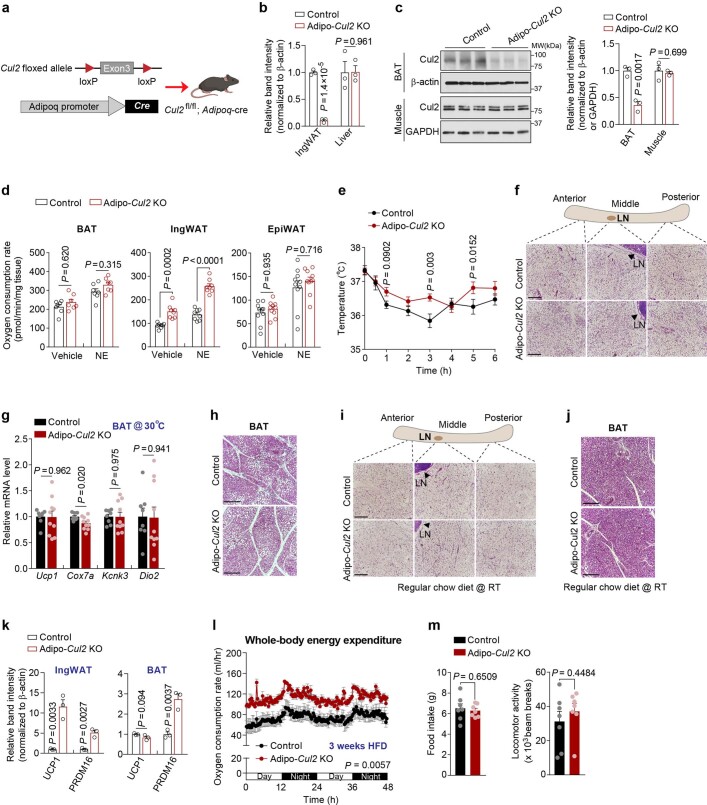
Characterization of Adipo-*Cul2* KO mice on a regular chow diet. **a**, Schematic illustration of adipocyte-selective *Cul2* knockout mice. The cartoon was created with Biorender. **b**, Quantification of Cul2 protein levels in Fig. 2a normalized to β-actin levels. *n* = 3 per group. **c**, Left: Immunoblotting of Cul2 protein in BAT and gastrocnemius muscle. The right panel shows the quantification normalized to β-actin or GAPDH. *n* = 3 per group. Representative results from two independent experiments. Gel source data are presented in Supplementary Fig. 1. **d**, Tissue OCR in Fig. 2c normalized by per mg of tissue. *n* = 7 for BAT in both groups, *n* = 8 for IngWAT in both groups, *n* = 10 for EpiWAT in both groups. **e**, Changes in rectal temperature of mice during adaptation to 8 °C from 30 °C. *n* = 7 per group. **f**, Representative H&E staining of inguinal WAT in **e**. LN, lymph node. **g**, Relative mRNA levels of indicated genes in iBAT of mice at 30 °C. *n* = 10 (Adipo-*Cul2* KO), *n* = 8 (Control). **h**, Representative H&E staining of iBAT in **e**. **i**, **j**, Representative H&E staining of inguinal WAT (**i**) and iBAT (**j**) of mice on a regular chow diet at room temperature. **k**, Quantification of indicated protein levels in Fig. 2d normalized to β-actin levels. *n* = 3 per group. **l**, Whole-body oxygen consumption rate of mice at 3 weeks of HFD. *n* = 7 for both groups. **m**, Total food intake and locomotor activity of mice in **l**. Representative images in **f**, **h**–**j** from two biologically independent samples. Scale bar, 210 μm. **b**–**e**, **g**, **k**–**m**, biologically independent samples. Data are mean ± s.e.m.; two-sided *P* values by unpaired Student’s *t*-test (**b**, **c**, **g**, **k**, **m**), one-way ANOVA followed by Tukey’s test (**d**) or two-way repeated-measures ANOVA (**l**) followed by Fisher’s LSD test (**e**). Source data

We next determined the extent to which the CUL2–RING E3 ligase in adipocytes regulates whole-body energy metabolism. To this end, we crossed *Cul2*^*flox/flox*^ mice with *Adipoq* (encoding adiponectin)-*cre* mice to generate fat-specific *Cul2*-knockout (*Adipoq*-*cre*;*Cul2*^*flox/flox*^, hereafter Adipo-*Cul2*-KO) mice (Fig. 2a and Extended Data Fig. 4a–c). Adipo-*Cul2-*KO mice expressed significantly higher levels of brown/beige-fat genes in the inguinal WAT relative to littermate controls (*Cul2*^*flox/flox*^) at 30 °C on a regular chow diet (Fig. 2b). Functionally, fatty acid oxidation and tissue respiration in the inguinal WAT, but not in the interscapular brown adipose tissue (iBAT) and gastrocnemius muscle, of Adipo-*Cul2*-KO mice were significantly higher compared with those of the controls when mice were exposed to 8 °C from 30 °C (Fig. 2c and Extended Data Fig. 4d). During cold exposure, Adipo-*Cul2*-KO mice exhibited modestly but significantly higher cold tolerance compared with the controls (Extended Data Fig. 4e). These changes were accompanied by the emergence of adipocytes containing multi-locular lipids in the inguinal WAT, although no noticeable difference was found in the iBAT (Extended Data Fig. 4f–j).

**Extended Data Fig. 5:**
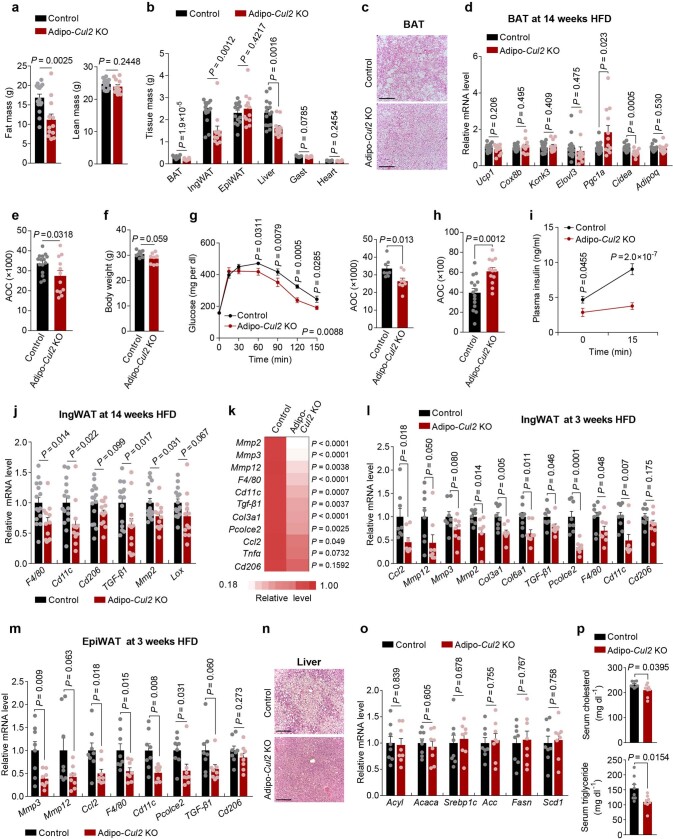
Adipo-*Cul2* KO mice on a HFD. **a**, Fat mass and lean mass at 8 weeks of HFD. *n* = 12 (Adipo-*Cul2* KO), *n* = 15 (Control). **b**, Tissue weight at 14 weeks of HFD. *n* = 12 (Adipo-*Cul2* KO), *n* = 15 (Control). **c**, Representative H&E staining of the iBAT at 14 weeks of HFD. Scale bar, 210 μm. **d**, Relative mRNA levels of indicated genes in the BAT. *n* = 12 (Adipo-*Cul2* KO), *n* = 15 (Control). **e**, Area of curve (AOC) of Fig. 2g was calculated by Graphpad software. **f**, Body weight at 3 weeks of HFD. *n* = 8 per group. **g**, Left: Glucose tolerance test at 3 weeks of HFD. *n* = 8 per group. *P*-value was determined by two-way repeated-measures ANOVA followed by Fisher’s LSD test. Right: AOC by Graphpad software. *P*-value was determined by unpaired Student’s *t*-test. **h**, AOC of Fig. 2h. **i**, Plasma insulin levels under fasted and glucose-stimulated conditions at 9 weeks of HFD. *n* = 12 (Adipo-*Cul2* KO), *n* = 15 (Control). **j**, Relative mRNA levels of pro-inflammatory genes and pro-fibrosis genes in the IngWAT at 14 weeks of HFD. *n* = 12 (Adipo-*Cul2* KO), *n* = 15 (Control). **k**, Heatmap of mRNA levels of indicated genes in the epididymal WAT at 14 weeks of HFD. *n* = 12 (Adipo-*Cul2* KO), *n* = 15 (Control). **l**, **m**, Relative mRNA levels of indicated genes in the IngWAT (**l**) and EpiWAT (**m**) at 3 weeks of HFD. *n* = 8 per group. **n**, Representative H&E staining of the liver at 14 weeks of HFD. Scale bar, 210 μm. **o**, Relative mRNA levels of indicated hepatic genes at 3 weeks of HFD. *n* = 8 per group. **p**, Serum cholesterol and triglyceride levels at 3 weeks of HFD. *n* = 8 per group. Representative images in **c** and **n** from two biologically independent samples per group. **a**, **b**, **d**–**m**, **o**, **p**, biologically independent samples. Data are mean ± s.e.m.; two-sided *P* values by unpaired Student’s *t*-test (**a**, **b**, **d**–**f**, **h**, **j**–**m**, **o**, **p**) or two-way repeated-measures ANOVA followed by Fisher’s LSD test (**i**). Source data

On a 60% high-fat diet (HFD), the inguinal WAT of Adipo-*Cul2-*KO mice expressed higher levels of PRDM16 and UCP1 protein compared with the control mice (Fig. 2d and Extended Data Fig. 4k). The iBAT of Adipo-*Cul2*-KO mice also expressed higher levels of PRDM16 protein compared with the controls, but no difference in UCP1 expression was observed between the genotypes. Notably, Adipo-*Cul2*-KO mice displayed significantly higher whole-body energy expenditure (VO_2_) relative to littermate controls at 3 weeks of HFD, a time point at which body weight between the two groups had not yet diverged, although no change was seen in their food intake and locomotor activity (Fig. 2e and Extended Data Fig. 4l,m). In accordance with the principle of energy balance, Adipo-*Cul2*-KO mice gained significantly less body-weight compared with their littermate controls at 4 weeks of HFD and thereafter owing to decreased fat mass (Fig. 2f and Extended Data Fig. 5a,b). By the end of the HFD studies, the iBAT of Adipo-*Cul2*-KO mice had fewer lipid droplets compared with in the iBAT of the controls, but there was no difference in thermogenesis gene expression (Extended Data Fig. 5c,d). Adipo-*Cul2*-KO mice exhibited significantly higher systemic glucose tolerance compared with their littermate controls (Fig. 2g and Extended Data Fig. 5e). Notably, this improved glucose tolerance was not merely a metabolic consequence of lower body weight, as Adipo-*Cul2*-KO mice already displayed higher glucose tolerance within 3 weeks of HFD when the body weight between the genotypes was not yet different (Extended Data Fig. 5f,g). Moreover, Adipo-*Cul2-*KO mice displayed higher insulin sensitivity compared with the control mice, as shown by insulin-tolerance tests, pyruvate-tolerance tests and glucose-stimulated insulin secretion tests (Fig. 2h,i and Extended Data Fig. 5h,i).

Note that adipose tissue fibrosis and inflammation were significantly repressed in the inguinal and epidydimal WAT of Adipo-*Cul2*-KO mice relative to the control mice at 3 weeks of HFD and thereafter (Extended Data Fig. 5j–m), supporting the recent observations that enhanced beige fat biogenesis is tightly coupled with reduced adipose tissue inflammation independent of UCP1^18,19^. The liver of Adipo*-Cul2*-KO mice contained fewer lipid droplets and triglycerides compared with their littermate controls, although we found no difference in the expression of de novo lipogenesis genes (Fig. 2j and Extended Data Fig. 5n,o). Furthermore, serum levels of cholesterol and triglycerides in Adipo-*Cul2-*KO mice were lower compared with the controls at 3 weeks of HFD and thereafter up to 14 weeks of HFD (Fig. 2k and Extended Data Fig. 5p).

## APPBP2 bridges CUL2 and PRDM16

**Fig. 3:**
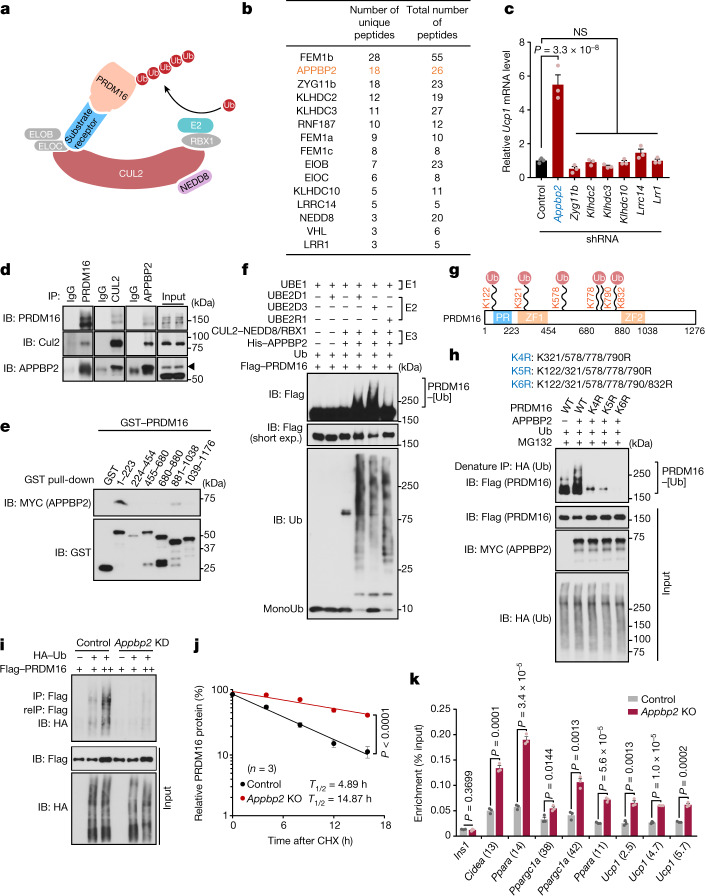
APPBP2 is the substrate receptor in the CUL2 complex that catalyses the polyubiquitination of PRDM16. **a**, A model of PRDM16 protein polyubiquitination by the CUL2–RING E3 ligase complex. **b**, Candidates of the substrate receptor in the CUL2 complex in adipocytes, HEK293T cells and myoblasts. APPBP2 is highlighted in orange. **c**, Relative mRNA levels of *Ucp1* in differentiated inguinal adipocytes expressing a scrambled control shRNA (control) or shRNAs targeting substrate receptor candidates. *Appbp2* is highlighted in blue. **d**, Endogenous protein interaction between PRDM16, CUL2 and APPBP2 in differentiated inguinal adipocytes treated with MG132. Inputs are shown on the right. IB, immunoblot; IP, immunoprecipitation. **e**, In vitro binding assay of purified GST-tagged PRDM16 fragments and Myc-tagged APPBP2. **f**, In vitro polyubiquitination of purified PRDM16 protein. **g**, Ubiquitination sites in PRDM16 protein. Immunoblotting data and MS data are provided in Extended Data Fig. 7b,c. **h**, PRDM16 polyubiquitination in HEK293T cells expressing the WT and the indicated mutant forms of PRDM16 in the presence of MG132. **i**, Changes in PRDM16 polyubiquitination in HEK293T cells expressing a scrambled control (control) or shRNA targeting *Appbp2* (*Appbp2* KD) in the presence of MG132. reIP, reimmunoprecipitation. **j**, Changes in endogenous PRDM16 protein stability in control and *Appbp2*-KO inguinal adipocytes. Immunoblotting data are provided in Extended Data Fig. 8a. **k**, ChIP–quantitative PCR (ChIP–qPCR) analysis of PRDM16 recruitment onto the target loci of PRDM16 in control and *Appbp2-*KO inguinal adipocytes. *Ins1* was used as a non-specific binding site. For **d**–**f**, **h**, **i**, representative results are shown from two independent experiments. Gel source data are presented in Supplementary Fig. 1. For **c**, **j** and **k**, *n* = 3 biologically independent samples per group. For **c**, **j** and **k**, data are mean ± s.e.m. Two-sided *P* values were calculated using one-way ANOVA followed by Dunnett’s test (**c**), two-way ANOVA (**j**) or unpaired Student’s *t*-tests (**k**). Source data

**Extended Data Fig. 6:**
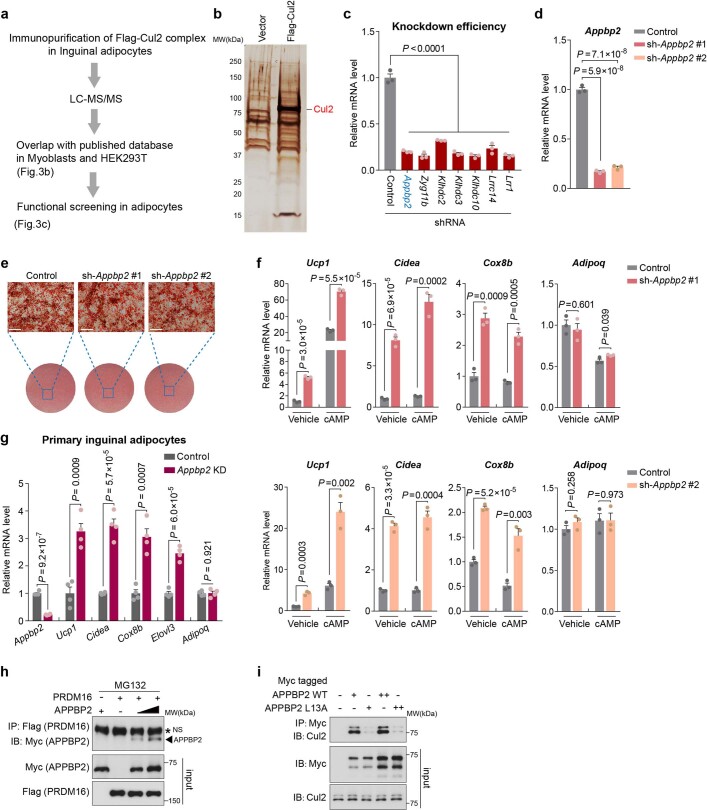
Depletion of *Appbp2* promotes fat thermogenesis. **a**, The experimental scheme for identifying the substrate receptor for PRDM16. **b**, Silver-stained gel of immuno-purified Flag-Cul2 complex in inguinal adipocytes. **c**, Relative mRNA levels of indicated genes in differentiated inguinal adipocytes expressing a scrambled control shRNA (Control) or shRNAs targeting respective substrate receptors. *n* = 3 per group. **d**, Relative mRNA levels of *Appbp2* in inguinal adipocytes stably expressing a scrambled control shRNA (Control) or two distinct shRNAs targeting *Appbp2* (#1, #2). *n* = 3 per group. **e**, Oil-Red-O staining of inguinal adipocytes stably expressing a scrambled control shRNA (Control) or shRNAs targeting *Appbp2* (#1, #2). Scale bar, 200 μm. **f**, Relative mRNA levels of indicated genes in inguinal adipocytes stably expressing a scrambled control shRNA (Control) or shRNAs targeting *Appbp2* (Upper: sh-*Appbp2* #1; Lower: sh-*Appbp2* #2) in the presence or absence of forskolin (cAMP). *n* = 3 per group. **g**, Relative mRNA levels of indicated genes in control and *Appbp2* KD primary inguinal adipocytes. *n* = 4 per group. **h**, The protein interaction between APPBP2 and PRDM16. *NS, non-specific band. **i**, The protein interaction between Cul2 and wild-type (WT) or L13A mutant form of APPBP2. Representative results in **b**, **e**, **h**, **i** from two independent experiments. Gel source data are presented in Supplementary Fig. 1. **c**, **d**, **f**, **g**, biologically independent samples. Data are mean ± s.e.m.; two-sided *P* values by one-way ANOVA followed by Dunnett’s test (**c**, **d**) or unpaired Student’s *t*-test (**f**, **g**). Source data

CUL2 functions as a scaffold protein by interacting with an E2 enzyme, elongin B (ELOB), elongin C (ELOC), and substrate-specific receptors (Fig. 3a). To identify a specific substrate receptor to PRDM16 protein, we devised the following strategy (Extended Data Fig. 6a). First, we immunopurified a CUL2 complex in differentiated beige adipocytes and analysed it using liquid chromatography coupled with tandem mass spectrometry (LC–MS/MS) (Extended Data Fig. 6b). Second, we overlapped the proteomics data with published datasets of CUL2-interacting proteins in HEK293T cells and myoblasts^23,24^, and identified 12 potential substrate receptors and CUL2-interacting subunits (Fig. 3b). Subsequently, we used short hairpin RNA (shRNA)-based screening to determine which substrate receptor(s) controlled *Ucp1* transcription (Extended Data Fig. 6c). Among these candidates, shRNA-mediated depletion of APPBP2, but not others, significantly increased *Ucp1* mRNA levels in differentiated adipocytes (Fig. 3c). We further validated the result in independent experiments in which depletion of APPBP2 using two distinct shRNAs in immortalized or primary inguinal WAT-derived adipocytes led to elevated expression of brown/beige-fat-selective genes without altering adipogenesis (Extended Data Fig. 6d–g).

**Extended Data Fig. 7:**
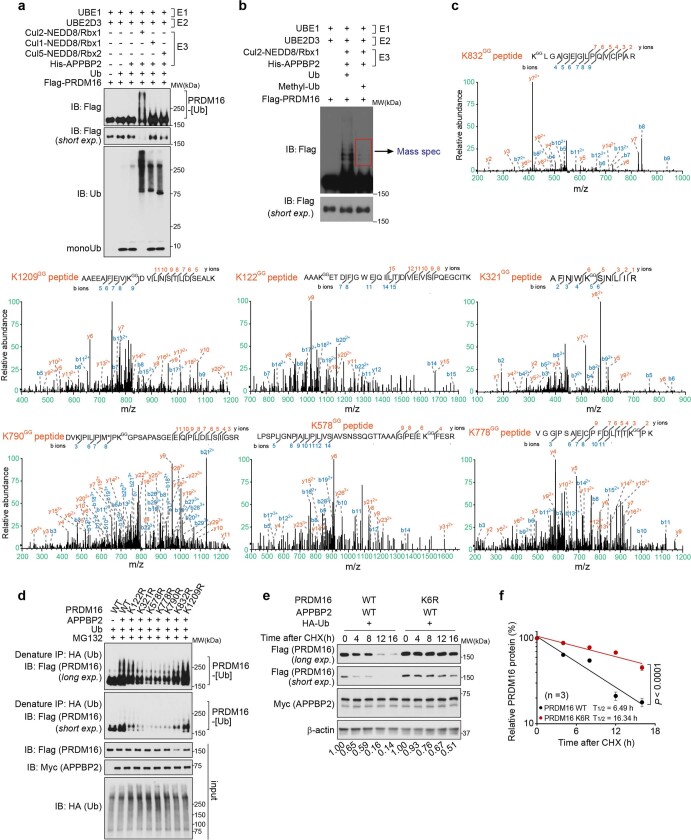
Cul2^APPBP2^ E3-ligase complex catalyses the polyubiquitination of PRDM16. **a**, Reconstitution of PRDM16 polyubiquitination by indicated purified proteins. **b**, *In vitro* mono-ubiquitination of purified PRDM16 protein. Ubiquitinated PRDM16 was detected by immunoblotting with an anti-Flag antibody and subsequently analysed by LC-MS/MS. **c**, Identification of ubiquitin sites on PRDM16 by mass spectrometry. The K^GG^ indicates that the lysine residue is modified by the addition of a di-glycine remnant. Predicted b- and y-type ions, are listed above and below the peptide sequence, respectively. Ions observed are labelled above the corresponding ion peaks in the spectrum. Seven sites of lysine ubiquitination were mapped to PRDM16. **d**, Polyubiquitination of the wild-type and indicated mutant forms of PRDM16. **e**, Changes in protein stability of the wild-type and ubiquitin-deficient form (K6R) of PRDM16. β-actin was used as a loading control. **f**, Expression levels of PRDM16 in **e** were quantified by Image J software and normalized to β-actin levels. *n* = 3 per group, biologically independent samples. Data are mean ± s.e.m.; two-sided *P* values by two-way ANOVA. Representative results in **a**, **b**, **d**, **e** from two independent experiments. Gel source data are presented in Supplementary Fig. 1. Source data

Whereas APPBP2 was originally identified as an amyloid precursor protein-binding protein^25^, APPBP2 was also found in RING E3 ligase complexes^26,27^. However, the pathophysiological role of APPBP2, remained unclear. Thus, we performed the following experiments to test the hypothesis that CUL2–APPBP2 catalyses the polyubiquitination of PRDM16 protein. First, we validated the interaction between PRDM16 and CUL2–APPBP2 proteins in inguinal WAT-derived adipocytes in the presence of the proteasome inhibitor MG132 (Fig. 3d and Extended Data Fig. 6h). APPBP2 interacted with CUL2 through interactions with ELOB and ELOC because a point mutant of APPBP2 (L13A), which lacked the binding interface to ELOB and ELOC^28^, failed to interact with CUL2 (Extended Data Fig. 6i). APPBP2 interacted directly with PRDM16 in a cell-free condition under which purified APPBP2 bound to two domains (amino acids 1–223 and 881–1038) of the PRDM16 protein (Fig. 3e). Second, we reconstituted the polyubiquitination reaction of PRDM16 by adding purified recombinant PRDM16 and the NEDD8-modified CUL2–APPBP2–RBX1 complex (E3), both of which were generated in the baculovirus expression system, in the presence of purified E1 ubiquitin-activating enzyme (UBE1), E2 ubiquitin-conjugating enzymes (UBE2D1, UBE2D3 or UBE2R1) and ubiquitin (Fig. 3f). This reaction is specific to the CUL2–APPBP2 complex because replacing CUL2 with CUL1 or CUL5 failed to trigger the polyubiquitination reaction of PRDM16 (Extended Data Fig. 7a). Third, using MS and point-mutation analyses of PRDM16 protein, we identified six biologically relevant ubiquitination sites of PRDM16 at Lys122, Lys321, Lys578, Lys778, Lys790 and Lys832 (Fig. 3g and Extended Data Fig. 7b–d). Importantly, Lys-to-Arg substitutions of these six ubiquitination sites (K6R) abolished PRDM16 protein ubiquitination and significantly extended protein half-life relative to the WT (Fig. 3h and Extended Data Fig. 7e,f).

**Extended Data Fig. 8:**
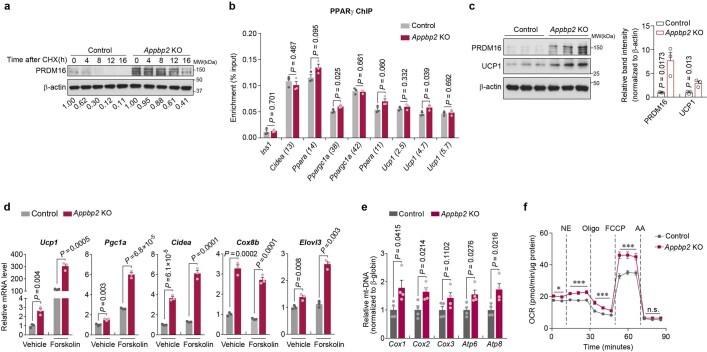
APPBP2 loss promotes PRDM16 protein stability and beige adipocyte biogenesis. **a**, Changes in endogenous PRDM16 protein stability in control and *Appbp2* KO inguinal adipocytes. PRDM16 protein levels were quantified by Image J software and normalized to β-actin levels. **b**, ChIP-qPCR analysis of PPARγ recruitment onto the PRDM16’s target loci in control and *Appbp2* KO inguinal adipocytes. *Ins1* was used as a nonspecific binding site. *n* = 3 per group. **c**, Left: Immunoblotting of indicated proteins in control and *Appbp2* KO inguinal adipocytes. Right: Protein quantification normalized to β-actin levels. *n* = 3 per group. **d**, Relative mRNA levels of indicated genes in control and *Appbp2* KO inguinal adipocytes in the presence or absence of forskolin. *n* = 3 per group. **e**, Mitochondrial DNA transcripts normalized to β-globin. *n* = 4 per group. **f**, OCR in control and *Appbp2* KO adipocytes. OCR values were normalized by total protein (μg). *n* = 10 (*Appbp2* KO), *n* = 9 (Control). Representative results in **a** and **c** from two independent experiments. Gel source data are presented in Supplementary Fig. 1. **b**–**f**, biologically independent samples. Data are mean ± s.e.m.; two-sided *P* values by unpaired Student’s *t*-test (**b**–**e**) or two-way repeated-measures ANOVA (**f**). * *P* < 0.05, *** *P* < 0.001. n.s., not significant. Exact *P*-values are in Supplementary Table 2. Source data

Next, we determined the extent to which APPBP2 controls endogenous PRDM16 protein stability and the recruitment onto the target genes in adipocytes. We found that depletion of *Appbp2* reduced polyubiquitination of PRDM16 and significantly extended the half-life of PRDM16 protein (Fig. 3i,j and Extended Data Fig. 8a). Chromatin-immunoprecipitation (ChIP) experiments further showed that *Appbp2* deletion enhanced the recruitment of PRDM16 onto all of the target genes that we examined, whereas the recruitment of PPARγ, which forms a complex with PRDM16, at the same target loci was not affected (Fig. 3k and Extended Data Fig. 8b). These data indicate that APPBP2 is the substrate receptor in the CUL2–RING E3 ligase complex that catalyses PRDM16 polyubiquitination and degradation.

## APPBP2 loss activates beige fat through PRDM16

**Fig. 4:**
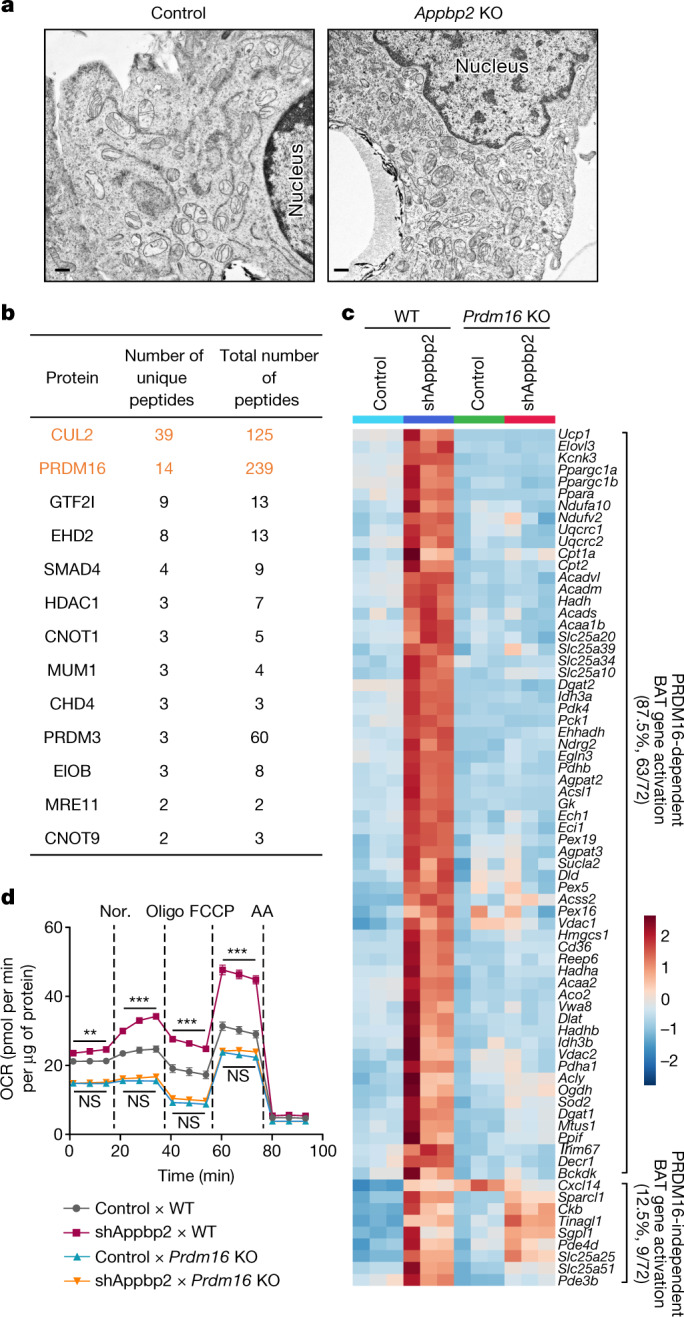
APPBP2 loss promotes beige adipocyte biogenesis through PRDM16. **a**, Cellular morphology of control and *Appbp2-*KO adipocytes by electron microscopy. Scale bar, 500 nm. Representative images from two biologically independent samples per group. **b**, Substrate candidates of APPBP2 in adipocytes. Cul2 and PRDM16 are highlighted in orange. **c**, Heat map of the RNA-seq transcriptome in WT or *Prdm16*-KO adipocytes expressing a scrambled control (control) or shRNA targeting *Appbp2* (shAppbp2) in the presence or absence of forskolin. *n* = 3 biologically independent samples per group. All of the listed genes are significantly different (FDR < 0.05) on the basis of analysis using edgeR. **d**, The OCR in inguinal adipocytes from WT and *Prdm16*-KO mice. *n* = 8 (control × WT), *n* = 10 (shAppbp2 × WT), *n* = 9 (control × *Prdm16* KO), *n* = 9 (shAppbp2 × *Prdm16* KO) biologically independent samples. Data are mean ± s.e.m. Two-sided *P* values were calculated using two-way repeated-measures ANOVA followed by Tukey test. **P < 0.01, ***P < 0.001. Source data

The results above led us to test the hypothesis that adipocyte-specific inhibition of APPBP2, as for CUL2, promotes beige fat biogenesis. To this end, we developed mice carrying a floxed *Appbp2* allele, from which inguinal WAT-derived adipose stromal fractions were isolated. Consistent with the results of *Cul2* deletion, we found that deletion of *Appbp2* led to higher PRDM16 protein levels compared with in the control cells, which was accompanied by increased expression of brown/beige-fat-selective genes, particularly when stimulated with forskolin (Extended Data Fig. 8c,d). *Appbp2*-KO adipocytes contained crista-rich mitochondria and expressed higher levels of mitochondria-encoded genes relative to control adipocytes (Fig. 4a and Extended Data Fig. 8e). Moreover, differentiated *Appbp2*-KO adipocytes exhibited significantly higher levels of total and uncoupled respiration than controls following noradrenaline treatment (Extended Data Fig. 8f).

**Extended Data Fig. 9:**
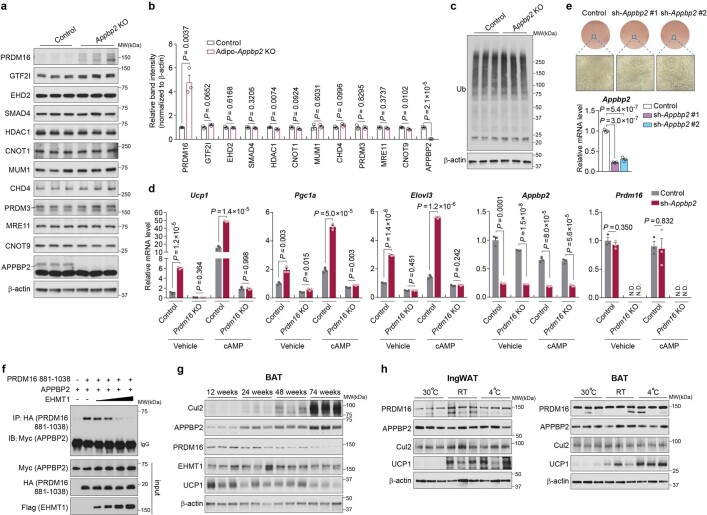
APPBP2 loss promotes adipocyte thermogenesis via PRDM16. **a**, Immunoblotting of indicated endogenous protein levels in control and *Appbp2* KO inguinal adipocytes. β-actin was used as a loading control. **b**, Quantification of indicated proteins in **a** normalized to β-actin levels. **c**, Global ubiquitin level in control and *Appbp2* KO inguinal adipocytes. **d**, Relative mRNA levels of indicated genes in control and *Prdm16* KO inguinal adipocytes expressing a scrambled control (Control) or shRNA targeting *Appbp2* (sh-*Appbp2*). N.D., not detected. **e**, Upper: Oil-Red-O staining of C2C12 myoblasts expressing a scrambled control (Control) or shRNAs targeting *Appbp2*. Lower: Relative *Appbp2* mRNA levels in C2C12 myoblasts. **f**, Protein interaction between PRDM16 (aa. 881–1038) and APPBP2 in the presence and absence of EHMT1. **g**, Immunoblotting of indicated proteins in the interscapular BAT of mice at 12, 24, 48, and 74 weeks old. **h**, Expression of indicated proteins in the inguinal WAT and interscapular BAT of 12-weeks-old mice at 30 °C for 2 weeks, at room temperature, and at 4 °C for 24 h. Representative results in **a**, c, **f**-**h** from two independent experiments. Gel source data are presented in Supplementary Fig. 1. **a**–**e**, **g**, **h**, *n* = 3 per group, biologically independent samples. Data are mean ± s.e.m.; two-sided *P* values by unpaired Student’s *t*-test (**b**, **d**) or one-way ANOVA followed by Dunnett’s test (**e**). Source data

To determine the substrate specificity of CUL2–APPBP2, we next performed three complementary experiments. First, we immunopurified the APPBP2 complex from beige adipocytes in the presence of MG132. The subsequent LC–MS/MS-based proteomics analysis identified PRDM16 as the most abundant protein and CUL2 as the second most abundant protein in the APPBP2 complex, as measured by total peptide number (Fig. 4b). These results verified the dominant interaction between PRDM16 and CUL2–APPBP2 in adipocytes. Although far less abundant than PRDM16 and CUL2, several other proteins also co-purified with APPBP2, such as GTF2I, EHD2, SMAD4 and PRDM3. However, none of these proteins were altered by APPBP2 loss, whereas PRDM16 protein expression was significantly higher in *Appbp2-*KO adipocytes compared with in the controls (Extended Data Fig. 9a,b). No change in global ubiquitin levels was observed in *Appbp2*-KO adipocytes (Extended Data Fig. 9c). Second, we systematically determined the functional relationship between PRDM16 and APPBP2 by performing transcriptomics analysis of adipocytes from *Prdm16*-KO mice and control mice lacking APPBP2. The analysis found that APPBP2 loss upregulated the expression of 72 genes that were known to be enriched in brown/beige adipocytes relative to white fat^29,30^. Among the 72 brown/beige-fat-selective genes, 87.5% (63 out of 72 genes) were upregulated in a PRDM16-dependent manner (Fig. 4c and Extended Data Fig. 9d). Another cellular model of PRDM16-dependent action is the myoblasts-to-brown-fat switch^5,6^, whereby depletion of *Appbp2* in C2C12 myoblasts, which lacked endogenous PRDM16, was insufficient to induce brown adipocyte differentiation (Extended Data Fig. 9e). Third, an analysis of cellular respiration found that *Appbp2* depletion significantly elevated total and uncoupled OCR in a PRDM16-dependent manner (Fig. 4d).

## APPBP2 controls whole-body metabolism

**Fig. 5:**
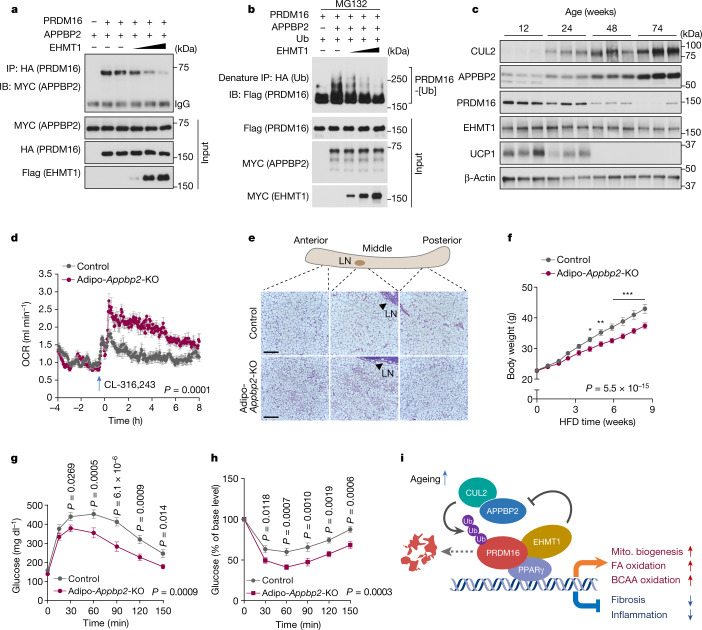
APPBP2 controls whole-body energy metabolism. **a**, Changes in the PRDM16–APPBP2 interaction in the presence of EHMT1. **b**, Changes in PRDM16 polyubiquitination in the presence of EHMT1. **c**, Immunoblot analysis of the indicated proteins in the inguinal WAT of mice aged 12, 24, 48 and 74 weeks. β-Actin was used as the loading control. *n* = 3 per group. **d**, The whole-body OCR of Adipo-*Appbp2-*KO and littermate control mice on a regular chow diet at 30 °C in response to treatment with CL-316,243. *n* = 8 for both groups. **e**, H&E staining of the inguinal WAT (anterior, middle and posterior regions) in Adipo-*Appbp2-*KO and control mice in **d**. Scale bars, 210 μm. Representative images from two biologically independent samples per group. LN, lymph node. **f**, The body weight of mice on an HFD. *n* = 10 (Adipo-*Appbp2*-KO) and *n* = 11 (control). **g**, Glucose-tolerance test of mice at 9 weeks of feeding on an HFD. **h**, Insulin-tolerance test of mice at 10 weeks of feeding on an HFD. **i**, A model of how CUL2–APPBP2 controls PRDM16 protein stability and beige fat biogenesis. The CUL2–APPBP2 E3 ligase complex catalyses polyubiquitination of PRDM16. Inhibition of CUL2–APPBP2 potently extends the protein half-life of PRDM16, leading to the activation of brown/beige fat genes as well as the repression of pro-inflammatory and pro-fibrosis genes in adipocytes. CUL2–APPBP2 expression in adipose tissues increases with age, showing an inverse correlation with the age-associated decline in PRDM16 protein. EHMT1 stabilizes PRDM16 protein by blocking the PRDM16–APPBP2 interaction. Mito., mitochondrial. For **a**–**c**, representative results are shown from two independent experiments. Gel source data are presented in Supplementary Fig. 1. For **c**, **d** and **f**–**h**, data are from biologically independent samples. For **d**, **f**, **g** and **h**, data are mean ± s.e.m. Two-sided *P* values were calculated using two-way repeated-measures ANOVA (**d** and **f**–**h**) followed by Fisher’s least significant difference test (**f**–**h**). *P < 0.05, **P < 0.01, ***P < 0.001. Exact *P* values are shown in Supplementary Table 2. Source data

Identification of CUL2–APPBP2 as the ubiquitin E3 ligase for PRDM16 enabled us to examine the regulatory mechanisms of PRDM16 protein stability. We and others previously reported that EHMT1 controls PRDM16 protein stability and brown/beige adipocyte development^14,31^. Here we found that EHMT1 displaced APPBP2 from the PRDM16 complex in a dose-dependent manner through the binding interface (amino acids 881–1038) of PRDM16 (Fig. 5a and Extended Data Fig. 9f). Importantly, the displacement of APPBP2 from the PRDM16 complex by EHMT1 recruitment reduced the polyubiquitination of PRDM16 protein (Fig. 5b). Another biological context of PRDM16 protein stability is ageing^18,32^ (Extended Data Fig. 1a,b): we found that APPBP2 and CUL2 expression in the inguinal WAT was increased at 24 weeks old and thereafter, correlating well with the age-associated decline in PRDM16 and UCP1 protein expression (Fig. 5c). Similarly, the expression of APPBP2 and CUL2 in the iBAT increased at 48 weeks old and thereafter (Extended Data Fig. 9g). By contrast, acute cold exposure changed neither PRDM16 nor CUL2–APPBP2 protein expression (Extended Data Fig. 9h).

**Extended Data Fig. 10:**
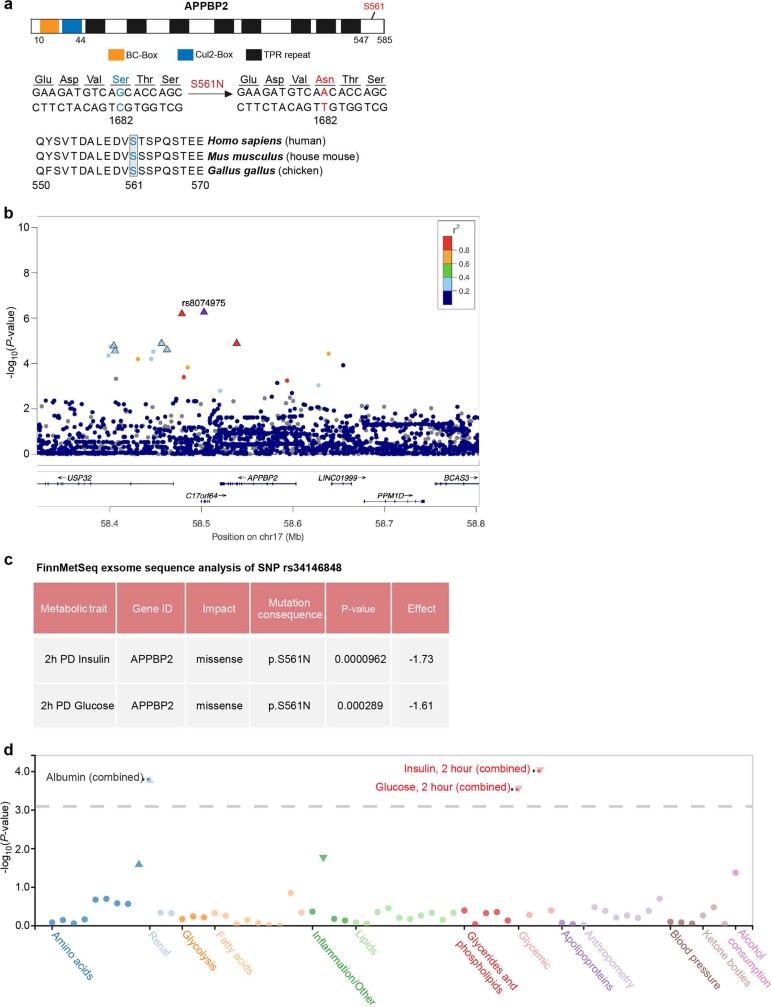
Association between human *APPBP2* variants and metabolic health. **a**, Upper: The linear domain structure of APPBP2. Ser 561 is indicated. Middle: The rs34146848 SNP causes a Ser561 to Asn mutation in the APPBP2 protein. Lower: The amino acid Ser 561 is evolutionally conserved among indicated vertebrates. **b**, LocusZoom regional association plot between SNPs in the *APPBP2* gene locus and waist-hip ratio adjusted for BMI in individuals of African descent. X-axis represents chromosomal location. Y-axis represents -log_10_ (*P*-value). Each point is an SNP coloured by linkage disequilibrium correlation with the lead SNP (rs8074975). SNPs denoted by triangles are in the 95% credible set of the association. **c**, *APPBP2* rs34146848 variant is significantly associated with lower levels of 2-h postprandial (PD) glucose and 2-h postprandial (PD) insulin. The data are obtained from the FinnMetSeq exome sequence analysis. **d**, The Phenome-wide association (PheWAS) plot shows the significant (*P* < 0.05) associations of 17:58525018:C:T/rs34146848 for all available traits, generated by bottom-line meta-analysis across all datasets in the Common Metabolic Diseases Knowledge Portal (CMDKP).

**Extended Data Fig. 11:**
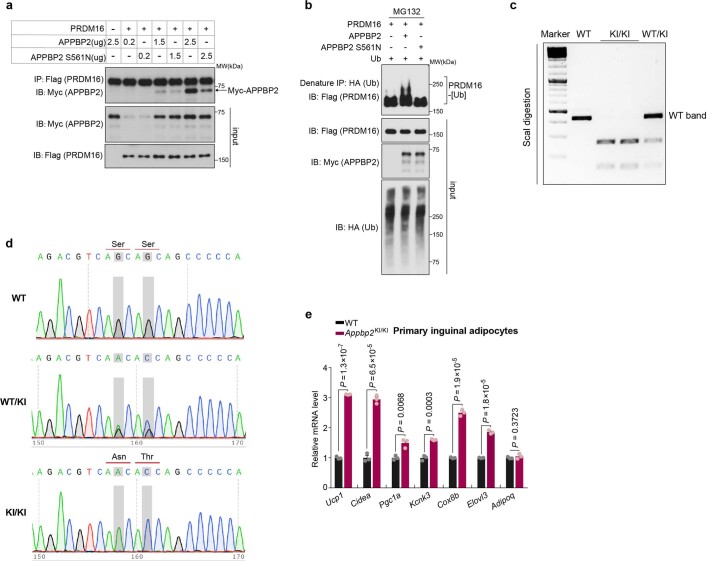
Functional characterization of the human APPBP2 S561N mutant. **a**, Protein interaction between PRDM16 and the wild-type (WT) or S561N mutant form of APPBP2. **b**, PRDM16 polyubiquitination catalysed by the wild-type (WT) or S561N mutant form of APPBP2. **c**, Genotyping of S561N knock-in mice that carried the S561N mutation in mouse *Appbp2* gene by PCR followed by enzymatic digestion with ScaI. **d**, Sanger sequencing of S561N knock-in mice. Note that the amino acid sequence of APPBP2 is well conserved between mice and humans in the C-terminus with the exception at T562 (S562 for mouse APPBP2, see Extended Data Fig. 10a). To recapitulate the human S561N variant of APPBP2 in mice, we introduced mutations at S561N and S562T of mouse APPBP2. **e**, Relative mRNA levels of indicated genes in primary inguinal WAT-derived adipocytes from S561N knock-in mice and control (WT) mice. Primary SVFs from the inguinal WAT were differentiated in culture. *n* = 3 per group, biologically independent samples. Data are mean ± s.e.m.; *P*-value was determined by two-tailed unpaired Student’s *t*-test. Representative results in **a**, **b**, from two independent experiments. Gel source data are presented in Supplementary Fig. 1. Source data

Several lines of evidence in human genetics imply a possible role of APPBP2 in metabolic health. First, the whole-exome sequencing data of 19,292 participants from FinnMetSeq exome sequencing data^33^ highlighted a single sequence variant (dbSNP: rs34146848) in the *APPBP2* gene that causes a Ser561 to Asn mutation in the APPBP2 protein at an evolutionally conserved amino acid residue among vertebrates (Extended Data Fig. 10a). As the single-nucleotide polymorphism (SNP) was relatively common in individuals of African ancestry^34^, we independently examined the genetic association at the *APPBP2* locus in an African ancestry cohort (*n* = 7,447) from the UK Biobank^35^. The analysis suggests an association between the *APPBP2* gene and waist–hip ratio adjusted for body mass index (lead SNP, rs8074975, *P* = 5.5 × 10^−7^), although no significant association with SNP rs34146848 was found in this cohort (Extended Data Fig. 10b). Regardless, there was a significant association between the APPBP2(S561N) variant and lower levels of 2 h postprandial serum glucose and insulin (Extended Data Fig. 10c,d), prompting us to further examine the degree to which the APPBP2(S561N) variant affects stability of PRDM16 protein. We found that APPBP2(S561N) displayed a weaker interaction with PRDM16 protein relative to the WT APPBP2 and nearly no polyubiquitination activity on PRDM16 protein (Extended Data Fig. 11a,b). Thus, we next generated CRISPR–Cas9-mediated *Appbp2* knock-in mice (*Appbp2*^*KI/KI*^) carrying the human S561N variant of APPBP2 by introducing mutations at S561N and S562T of mouse APPBP2 (Extended Data Figs. 10a and 11c,d). Differentiated primary inguinal WAT-derived adipocytes from *Appbp2*^*KI/KI*^ mice expressed significantly higher levels of brown/beige-fat-selective genes compared with WT adipocytes (Extended Data Fig. 11e).

**Extended Data Fig. 12:**
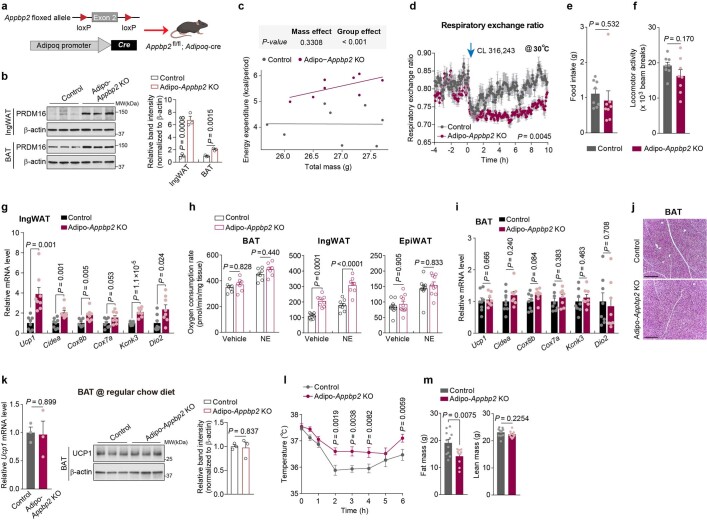
Characterization of Adipo-*Appbp2* KO mice. **a**, Schematic illustration of Adipo-*Appbp2* KO mice. The cartoon was created with Biorender. **b**, Left: Immunoblotting of indicated proteins. *n* = 3 per group. Right: Quantification of PRDM16 protein levels normalized to β-actin levels. *n* = 3 per group. **c**, Regression-based analysis of energy expenditure in Fig. 5d by CaIR-ANCOVA. **d**, Respiratory exchange ratio of mice in Fig. 5d. **e**, **f**, Total food intake (**e**) and locomotor activity (**f**) in Fig. 5d. **g**, Relative mRNA levels of indicated genes in the IngWAT in Fig. 5d. **h**, OCR in indicated tissues in Fig. 5d normalized by per mg of tissue. *n* = 7 for BAT in both groups, *n* = 8 for IngWAT in both groups, *n* = 10 for EpiWAT in both groups. **i**, Relative mRNA levels of indicated genes in the iBAT in Fig. 5d. *n* = 8 per group. **j**, H&E staining of iBAT in Fig. 5d. Scale bar, 210 μm. Representative images from two biologically independent samples. **k**, Left: Relative mRNA levels of *Ucp1* in the iBAT of mice on a regular chow diet at room temperature. *n* = 3 per group. Middle: Immunoblotting of UCP1 protein in the BAT. *n* = 3 per group. Right: Quantification of UCP1 protein levels normalized to β-actin levels. *n* = 3 per group. **l**, Changes in rectal temperature of mice during adaptation to 8 °C from 30 °C. *n* = 7 (Adipo-*Appbp2* KO), *n* = 6 (Control). **m**, Fat mass and lean mass at 8 weeks of HFD. *n* = 10 (Adipo-*Appbp2* KO), *n* = 11 (Control). Representative results in **b**, **k** from two independent experiments. Gel source data are presented in Supplementary Fig. 1. **b**-**i**, **k**-**m**, biologically independent samples. Data are mean ± s.e.m.; two-sided *P* values by unpaired Student’s *t*-test (**b**, **e**–**g**, **i**, **k**, **m**), one-way ANOVA followed by Tukey’s test (**h**) or two-way repeated-measures ANOVA (**d**) followed by Fisher’s LSD test (**l**). Source data

These results motivated us to characterize the metabolic phenotype of fat-specific *Appbp2*-KO mice (*Adipoq*-*cre*;*Appbp2*^*flox/flox*^, hereafter Adipo-*Appbp2-*KO) (Extended Data Fig. 12a). We confirmed that the inguinal WAT and iBAT of Adipo-*Appbp2*-KO mice expressed significantly higher levels of PRDM16 protein than those of the controls (Extended Data Fig. 12b). When mice were kept at 30 °C on a regular chow diet, the VO_2_ of Adipo-*Appbp2-*KO mice was significantly higher than that of their littermate controls after administration of CL-316,243 (Fig. 5d and Extended Data Fig. 12c). In turn, Adipo-*Appbp2*-KO mice exhibited a lower respiratory exchange ratio (VCO_2_/VO_2_) compared with the controls, while no difference was observed in their food intake and locomotor activity (Extended Data Fig. 12d–f). Notably, the inguinal WAT of Adipo-*Appbp2*-KO mice contained multilocular adipocytes and displayed significantly higher OCR and thermogenesis gene expression compared with those of control mice (Fig. 5e and Extended Data Fig. 12g,h). By contrast, we did not find noticeable differences in the iBAT and epidydimal WAT between the groups on a regular chow diet (Extended Data Fig. 12h–k). Adipo-*Appbp2-*KO mice displayed modestly high cold tolerance relative to the controls when mice were exposed to 8 °C from 30 °C (Extended Data Fig. 12l).

**Extended Data Fig. 13:**
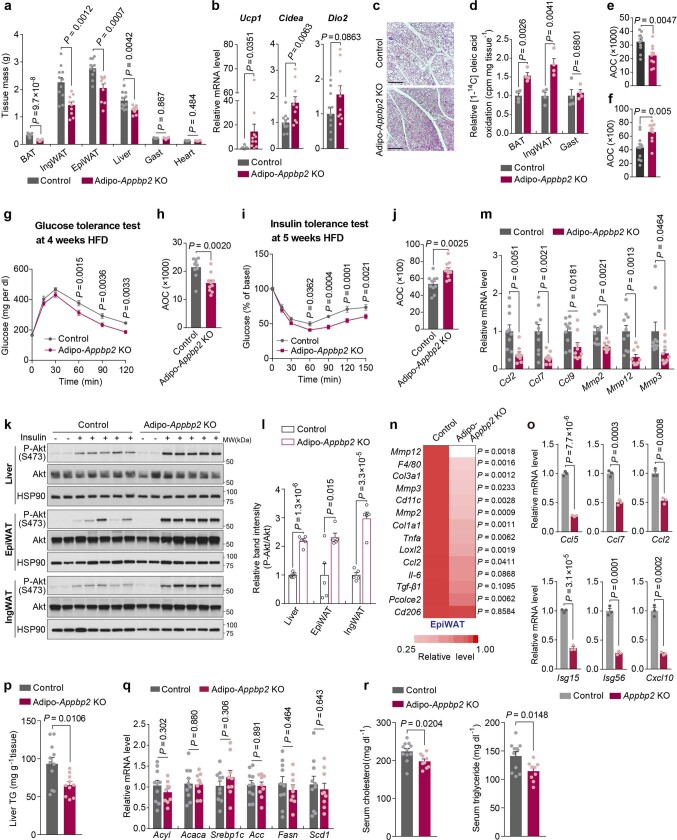
Metabolic profile of Adipo-*Appbp2* KO mice on HFD. **a**, Tissue weight at 12 weeks of HFD. *n* = 10 (Adipo-*Appbp2* KO), *n* = 11 (Control). **b**, Relative mRNA levels of indicated genes in the IngWAT of mice in Fig. 5f. **c**, H&E staining in the iBAT of mice in Fig. 5f. Scale bar, 210 μm. Representative images from two biologically independent samples per group. **d**, Oleic acid oxidation in the indicated tissues at 10 weeks of HFD. *n* = 4 per group. **e**, **f**, AOC of in Fig. 5g (**e**) and Fig. 5h (**f**). **g**, Glucose tolerance test at 4 weeks of HFD. *n* = 10 per group. **h**, AOC of **g**. **i**, Insulin tolerance test at 5 weeks of HFD. *n* = 10 per group. **j**, AOC of **i**. **k**, **l**, Immunoblotting of phosphorylated (S473) and total Akt protein in the liver, EpiWAT and IngWAT at 4 weeks of HFD. *n* = 7 (Adipo-*Appbp2* KO), *n* = 7 (Control) relative to HSP90 (**k**). Right: Quantification of phosphorylated (S473) AKT protein levels normalized to total AKT protein levels. *n* = 5 per group (**l**). **m**, Relative mRNA levels of indicated genes in the IngWAT at 4 weeks of HFD. *n* = 9 (Adipo-*Appbp2* KO), *n* = 10 (Control). **n**, Heat-map of indicated genes in the epididymal WAT at 12 weeks of HFD. **o**, Relative mRNA levels of indicated genes in Extended Data Fig. 8d. *n* = 3 per group. **p**, Hepatic TG content at 12 weeks of HFD. **q**, Relative mRNA levels of indicated hepatic genes at 4 weeks of HFD. *n* = 9 (Adipo-*Appbp2* KO), *n* = 10 (Control). **r**, Serum cholesterol and triglyceride levels in **p**. **a**, **b**, **d**–**r**, biologically independent samples. Data are mean ± s.e.m.; two-sided *P* values by unpaired Student’s *t*-test (**a**, **b**, **d**–**f**, **h**, **j**, **l**–**r**) or two-way repeated-measures ANOVA followed by Fisher’s LSD test (**g**, **i**). Source data

Next, we analysed the metabolic phenotype of Adipo-*Appbp2*-KO mice on an HFD under consistent conditions, including sex, age, diet and temperature, comparable to the studies of Adipo-*Cul2*-KO mice. At 4.2 weeks of HFD and thereafter, Adipo-*Appbp2-*KO mice gained significantly less body weight compared with the controls due to lower adipose tissue mass (Fig. 5f and Extended Data Fig. 12m). At 12 weeks of HFD, the inguinal WAT of Adipo-*Appbp2-*KO mice had significantly lower tissue mass and expressed higher levels of *Ucp1*, *Cidea* and *Dio2* relative to the control mice (Extended Data Fig. 13a,b). As seen in Adipo-*Cul2*-KO mice, the iBAT depots of Adipo-*Appbp2-*KO mice accumulated fewer lipid droplets than those of control mice (Extended Data Fig. 13c). Furthermore, fatty acid oxidation in the iBAT and inguinal WAT of Adipo-*Appbp2-*KO mice was significantly higher compared with in control mice on an HFD (Extended Data Fig. 13d).

Adipo-*Appbp2-*KO mice were more glucose tolerant and insulin sensitive relative to littermate controls after 9 and 10 weeks of HFD feeding, respectively (Fig. 5g,h and Extended Data Fig. 13e,f). Note that increased glucose tolerance and insulin sensitivity were already observed at early time points of HFD feeding when the body weight between the genotypes had not yet diverged (Extended Data Fig. 13g–l). Moreover, the expression levels of pro-inflammatory genes in the WAT of Adipo-*Appbp2*-KO mice were significantly lower compared with in controls at 4 weeks of HFD feeding and thereafter, and this occurred in a cell-autonomous manner (Extended Data Fig. 13m–o). The liver of Adipo-*Appbp2*-KO mice contained lower levels of triglycerides compared with the liver of the controls, although no difference was observed in hepatic lipogenic gene expression (Extended Data Fig. 13p,q). Finally, serum cholesterol and triglyceride levels in Adipo-*Appbp2-*KO mice were significantly lower compared with in the control mice (Extended Data Fig. 13r). Together, these results suggest that CUL2–APPBP2 deficiency in adipose tissues protects against diet-induced obesity, glucose intolerance, insulin resistance, hepatic steatosis and dyslipidaemia.

## Discussion

Our work suggests the following model of how beige adipocyte biogenesis is controlled through PRDM16 protein stability (Fig. 5i): the CUL2–APPBP2 ubiquitin E3 ligase complex catalyses the polyubiquitination and degradation of PRDM16 protein. Inhibition of CUL2–APPBP2 extends PRDM16 protein half-life, leading to the cell-autonomous activation of thermogenesis genes as well as the repression of pro-inflammatory/fibrotic genes in adipocytes. CUL2–APPBP2 expression in adipose tissues increases with age, showing an inverse correlation with the age-associated decline in PRDM16 protein expression independent of its mRNA expression. In turn, EHMT1 stabilizes PRDM16 protein by competing with APPBP2 for direct binding to PRDM16 and blocking PRDM16 polyubiquitination. How the CUL2–APPBP2–PRDM16 interaction is regulated in response to other pathophysiological cues, such as cachexia, hormonal cues and diet, is an important topic of future research.

Here we highlighted the post-translational control of beige fat biogenesis and the effect on metabolic health. However, we note that enhanced fatty acid oxidation in the BAT also contributed to the improved metabolic profile of fat-specific APPBP2-KO mice on an HFD. In agreement with emerging evidence regarding the UCP1-independent role of beige fat^18,19,36^, fat-specific CUL2–APPBP2 blockade not only improved glucose intolerance, insulin resistance and lipid profile, but also suppressed WAT inflammation and fibrosis before changes in body weight. Thus, it is probable that improved metabolic health, achieved by PRDM16 protein stabilization, extends far beyond UCP1-mediated thermogenesis and body-weight loss.

## Methods

### Animals

All of the animal experiments in this study were performed in compliance with protocols approved by the Institutional Animal Care and Use Committee (IACUC) at UCSF and Beth Israel Deaconess Medical Center. Unless otherwise specified, all of the mice had free access to food and water, and were housed under 12 h–12 h light–dark cycle, at 22 °C, and 45% humidity on average. *Cul2*^*flox/−*^ mice in the C57BL/6J background were generated by Applied StemCell using CRISPR–Cas9 technology. *Appbp2*^flox/−^ mice in the C57BL/6J background were made by Cyagen with CRISPR–Cas9. Adipocyte-specific *Cul2*-KO (Adipo-*Cul2*-KO mice) and *Appbp2*-KO mice (Adipo-*Appbp2-*KO mice) were developed by crossing *Cul2* or *Appbp2* floxed mice with *Adiponectin*-Cre mice (B6; FVB-Tg (*Adipoq*-Cre)1Evdr/J, 028020). *Appbp2* knock-in mice that carry the S561N mutation (equivalent to the human SNP in the *APPBP2* gene) were generated by Cyagen. To recapitulate the human S561N variant of *APPBP2* in mice, we mutated Ser561 to Asn and Ser562 to Thr by co-injecting the gRNA and the donor oligo containing p.Y551 (TAT to TAC) and p.A556 (GCC to GCG) into fertilized mouse oocytes. A list of the primer sequences used for genotyping and gRNA is provided in Supplementary Table 1.

### Cell culture

The stromal vascular fraction (SVF) from the inguinal WAT of C57BL/6J mice, *Prdm16*^*flox/flox*^ mice and *Appbp2*^*flox/flox*^ mice were immortalized by expressing the SV40 large T antigen as described previously^29^. For the generation of *Prdm16*-KO and *Appbp2*-KO cells, immortalized *Prdm16*^*flox/flox*^ or *Appbp2*^*flox/flox*^ preadipocytes were infected with retrovirus expressing *cre* (34565, Addgene), followed by hygromycin selection at a dose of 200 μg ml^−1^. For the generation of inguinal preadipocytes stably expressing *Cul2*, immortalized preadipocytes were infected with retrovirus expressing codon-optimized mouse *Cul2*, followed by blasticidin selection at a dose of 10 µg ml^−1^. For AAV infection of primary SVF cells, cells isolated from *Cul2*^*flox/flox*^ or *Appbp2*^*flox/flox*^ mice were infected with AAV-cre (105537-AAV8, Addgene) or AAV-GFP (105530-AAV8, Addgene) in growth medium overnight. After changing to a fresh growth medium, the cells were cultured for another 60 h. Preadipocytes were seeded into coated plates, and differentiation was induced by culturing cells with DMEM medium containing 10% FBS (Atlanta Biologicals), 5 µg ml^−1^ insulin, 1 nM T3, 0.5 µM rosiglitazone, 0.5 mM isobutylmethylxanthine, 125 nM indomethacin and 2 µg ml^−1^ dexamethasone. After 48 h, cells were cultured in medium containing 10% FBS, 5 µg ml^−1^ insulin, 1 nM T3 and 0.5 μM rosiglitazone for another 5–7 days. HEK293T cells, HEK293 virus packaging cells and C2C12 cells were maintained in DMEM high-glucose medium containing 10% FBS and 1% penicillin–streptomycin.

### Plasmids

*Cul1* (19896), *Cul2* (19892), *Cul3* (19893), *Cul4a* (19951), *Cul4b* (19922), *Cul5* (19895) and *Cul7* (20695) were obtained from Addgene. The EHMT1, HA-Ub, HA-PRDM16, Flag-PRDM16, GST-PRDM16 constructs were developed in our laboratory. Human *APPBP2* cDNA and mouse *Appbp2* cDNA constructs were amplified using standard PCR techniques from plasmids (MHS6278-202757182 for human *APPBP2*, MMM1013-202761267 for mouse *Appbp2*, Horizon) and subsequently inserted into mammalian expression vectors. Codon-optimized mouse *Cul2* cDNA and codon-optimized human *Appbp2* cDNA were synthesized by GenScript and cloned into a PMSCV-blasticidin vector (75085, Addgene). Point mutations used in the study were introduced using site-directed mutagenesis with the In-Fusion HD Cloning Plus (638909, Takara Bio) kit. All of the constructs were confirmed by sequencing.

### Antibodies and reagents

The following reagents were used in this study: MG132 (474790, Sigma-Aldrich), MLN4924 (501146629, Thermo Fisher Scientific), anti-Flag(R) M2 affinity gel (A2220, Sigma-Aldrich), Pierce anti-HA magnetic beads (88836, Thermo Fisher Scientific), 3×Flag peptide (F4799, Sigma-Aldrich), 3×HA peptide (AS-63764, Anaspec). The following antibodies were used in this study: anti-UCP1 (ab-10983, Abcam), anti-UCP1 (U6382, Sigma-Aldrich), anti-CUL2 (sc-166506, Santa Cruz), anti-recombinant CUL2 (EPR3104(2)) (ab166917, Abcam), anti-APPBP2 (NBP2-81781, Novus), anti-Flag-HRP (A8592, Sigma-Aldrich), anti-HA (sc-7392, Santa Cruz), anti-MYC (sc-40, Santa Cruz), anti-PPARγ (E-8) (sc-7273, Santa Cruz), OXPHOS cocktail (ab110413, Abcam), anti-ubiquitin (sc-8017, Santa Cruz), anti-GST (sc-138, Santa Cruz), anti-GAPDH (sc-32233, Santa Cruz), anti-β-actin (A3854, Sigma-Aldrich), anti-MUM1 (12682-1-AP, Proteintech), anti-SMAD4 (10231-1-AP, Proteintech), anti-EHD2 (11440-1-AP, Proteintech), anti-GTF2I (10499-1-AP, Proteintech), anti-HDAC1 (10197-1-AP, Proteintech), anti-CNOT1 (14276-1-AP, Proteintech), anti-CNOT9 (22503-1-AP, Proteintech), anti-CHD4 (14173-1-AP, Proteintech), anti-PRDM3 (C50E12) (2593S, Cell Signaling Technology), anti-EHMT1(E6Q8B) (35005S, Cell Signaling Technology), PhosphoPlus Akt (Ser473) Antibody Duet (8200S, Cell Signaling Technology), anti-HSP90 (4874S, Cell Signaling Technology), normal mouse IgG (sc-2025, Santa Cruz), rabbit IgG, polyclonal-isotype control (ab37415, Abcam), goat anti-rabbit light chain secondary antibody (NBP2-75935, Novus), goat anti-mouse IgG, light-chain specific antibody (91196S, Cell Signaling Technology), anti-PRDM16 (AF6295, R&D systems), polyclonal antibody against PRDM16 generated by immunizing rabbit with recombinant human PRDM16 (GenScript).

### shRNA constructs and virus production

The following lentiviral shRNA clones were purchased from GeneCopoeia: scrambled control (CSHCTR001-LVRH1H); *Cul2* (MSH036079-LVRH1H); *Klhdc2* (MSH034863- LVRH1H); *Lrrc14* (MSH038419-LVRH1H). Lentiviral shRNA constructs were generated by cloning into pLKO.1-hygromycin (24150, Addgene) or pLKO.1-blasticidin (26655, Addgene). A list of the sequences used in the study is provided in Supplementary Table 1.

For lentivirus production, HEK293 packaging cells were transfected with 10 µg lentiviral plasmids and 10 µg packaging plasmids (psPAX2 and pMD2.G) using the calcium phosphate method. After 48 h, the culture supernatant was collected and filtered using a 0.45 µm filter. Inguinal WAT-derived SVF cells or HEK293T cells were incubated with the viral supernatant supplemented with 10 µg ml^−1^ polybrene for 24 h. Subsequently, stable cell lines were obtained by selection with the indicated antibiotics: hygromycin B (10687010, Thermo Fisher Scientific) at a dose of 200 µg ml^−1^, puromycin (A1113803, Gibco) at 1 µg ml^−1^ or blasticidin (A1113903, Thermo Fisher Scientific) at 10 µg ml^−1^.

### Overexpression of *Cul2* and *Appbp2* in adipocytes

Flag-tagged codon-optimized mouse *Cul2* cDNA and HA-tagged codon-optimized human *Appbp2* cDNA were synthesized by GenScript and cloned into a PMSCV-blasticidin vector (75085, Addgene). For retrovirus production, HEK293 packaging cells were transfected with 10 µg above plasmid and 10 µg packaging plasmids (VSV and gag-pol) using the calcium phosphate method. After 48 h, the culture supernatant was collected and filtered by a 0.45 µm filter. Inguinal WAT-derived SVFs or *Appbp2*-KO preadipocytes were infected with retroviruses with 10 µg ml^−1^ polybrene for 24 h. Subsequently, cells were selected by blasticidin at a dose of 10 µg ml^−1^.

### Affinity purification of protein complex and proteomics

Protein complex purification was performed as previously described^4,6^. For CUL2 complex purification, immortalized preadipocytes derived from mouse inguinal WAT were infected with retrovirus expressing Flag-tagged *Cul2* or an empty vector. Adipocytes were grown to post-confluence and differentiated for 4 days. Cell extracts were prepared using lysis buffer (50 mM Tris-Cl pH 7.4, 150 mM NaCl, 1% Triton X-100, 1 mM EDTA) supplemented with a protease inhibitor cocktail (Roche). The supernatant from lysates was incubated with anti-Flag M2 affinity gel for 2 h. The immunoprecipitants were washed three times and subsequently eluted by 3×Flag peptide (Sigma-Aldrich). The eluants were then TCA-precipitated, separated in a 4–15% acrylamide gradient gel, and visualized by silver staining or Coomassie blue staining.

For APPBP2 complex characterization, immortalized *Appbp2*-KO preadipocytes derived from mouse inguinal WAT were infected with retrovirus expressing HA-tagged *Appbp2* or an empty vector. The adipocytes were grown to post-confluence and differentiated for 4 days, followed by MG132 treatment. The cells were homogenized to prepare nuclear extracts. The nuclear extracts were incubated with Pierce anti-HA magnetic beads (Thermo Fisher Scientific) for 2 h and then washed in a binding buffer (50 mM Tris-Cl pH 7.4, 150 mM NaCl, 0.5% Triton X-100). The complexes were eluted by 3×HA peptide (Anaspec), underwent TCA-precipitation, separated in a 4–15% acrylamide gradient gel, and subsequently visualized by silver staining or Coomassie blue staining.

Gel-resolved proteins were excised, digested with trypsin and individually analysed using reverse-phase liquid chromatography with tandem mass spectrometry (LC–MS/MS) using a high-resolution hybrid mass spectrometer (LTQ-Orbitrap, Thermo Fisher Scientific) with the TOP10 method at the Taplin Biological Mass Spectrometry Facility in Harvard Medical School. The LC–MS/MS data were searched against the IPI mouse database^37^. Proteins identified with at least two unique valid peptides were considered to be significant, and the FDR was estimated to be 0% using the target–decoy approach^38^.

### Protein expression and purification

SF9 cells were obtained from the UC Berkeley Cell Culture Facility. SF9 cells were cultured in Sf-900 II SFM (10902088, Thermo Fisher Scientific) supplemented with 1% penicillin–streptomycin (Gibco) and were maintained at 27 °C without CO_2_. Baculovirus packaging and amplification were performed according to the established commercial protocol of the Bac-to-Bac C-His TOPO Expression System (A11100, Thermo Fisher Scientific). In brief, C-terminally His-tagged *Appbp2* and *Flag*-*Prdm16* cDNAs were cloned into the pFastBac TOPO vector and transformed into DH10Bac *Escherichia coli* competent cells to form a recombinant expression bacmid. The bacmid was then transfected into SF9 cells using ExpiFectamine Sf Transfection Reagent for the production of recombinant baculovirus particles (P0 virus). The high titre baculovirus was amplified by infection of more insect cells with P0/P1 virus. For the purification, SF9 insect cells in 1 l medium were infected and collected by centrifugation and frozen at −80 °C. Cells were incubated and stirred with the lysis buffer (50 mM Tris-Cl pH 7.4, 150 mM KCl, 1 mM PMSF, 10 mM imidazole and 0.1% Triton X-100) supplemented with a protease inhibitor cocktail (Roche) using a stir bar at 4 °C for 1 h. Subsequently, cells were Dounce-homogenized 50 times, sonicated and then centrifuged at 26,000 rpm for 1 h. The supernatant was mixed with Ni-NTA slurry and bound for 1 h at 4 °C. Beads were washed in wash buffer (50 mM Tris-Cl pH 7.4, 150 mM KCl, 20 mM imidazole) three times for 15 min and eluted in a buffer containing 50 mM Tris-Cl pH 7.4, 150 mM KCl and 250 mM imidazole. Eluted proteins were exchanged into storage buffer (50 mM Tris-Cl pH 7.4, 100 mM KCl, 1 mM DTT, PMSF, 10% glycerol), aliquoted and flash-frozen.

### In vitro ubiquitination assays

Purified His–Flag–PRDM16 (400 nM), purified His–APPBP2 (600 nM), CUL2 (neddylated)/RBX1 (600 nM), or CUL1 (neddylated)/RBX1 (600 nM), or CUL5 (neddylated)/RBX2 (600 nM), UBE1 (120 nM), ubiquitin (20 µM), UBE2D1, or UBE2D3 or UBE2R1 (400 nM) were mixed in a reaction buffer containing 50 mM Tris–HCl, pH 7.4, 5 mM MgCl_2_, 2 mM ATP and 1 mM DTT. The reaction was carried out in a 30 μl volume at 37 °C for 60 min, and then resolved by SDS–PAGE. Ubiquitinated products were detected by immunoblotting. All of the proteins were obtained from Boston Biochem unless specified.

### Ubiquitination assays in cells

HEK293T cells were transfected with the indicated plasmids using the calcium phosphate method. After 42 h, 20 µM MG132 was directly added to the medium. After incubation for 6 h, the cells were collected and lysed with RIPA buffer (9806S, Cell Signaling Technology) containing 1% SDS supplemented with EDTA-free protease inhibitors. Cell lysates were briefly sonicated, boiled at 95 °C for 10 min, and then centrifuged at 12,000 rpm for 15 min. The supernatants were then diluted 1:9 with RIPA lysis buffer to reduce the SDS concentration to 0.1%. Pierce anti-HA magnetic beads (88837, Thermo Fisher Scientific) were incubated with diluted lysates for 2 h at 4 °C. Immunoprecipitates were washed four times with RIPA lysis buffer and analysed by immunoblotting.

### Identification of ubiquitination sites

Purified recombinant Flag–PRDM16 protein was processed for the in vitro ubiquitination reaction using methyl-Ub. The ubiquitinated Flag–PRDM16 was separated by SDS–PAGE, and the gel was stained with Coomassie blue. Protein bands were excised, destained and reduced in 1 mM DTT at 60 °C for 30 min, followed by alkylation in 5 mM iodoacetamide. After in-gel digestion, peptides were extracted from the gel with 5% formic acid/50% acetonitrile and dried completely in a speed-vac. At the time of analyses, samples were resuspended in a 2%/0.1% acetonitrile/formic acid solution. The extracted peptides were separated by an analytical capillary column (100 μm × 25 cm) packed with 2.6 μm spherical C18-reversed-phase silica beads (Accucore, Thermo Fisher Scientific). The Accela 600 HPLC pump was used to generate the following HPLC gradient: 5–35% in 60 min (A, 0.1% formic acid in water; B, 0.1% formic acid in acetonitrile). The eluted peptides were sprayed into a LTQ Orbitrap Velos Pro ion-trap mass spectrometer (Thermo Fisher Scientific) equipped with a nano-electrospray ionization source. The mass spectrometer was operated in data-dependent mode with one MS scan followed by 20 collision-induced dissociation for each cycle. Database searches were performed using Sequest (Thermo Fisher Scientific) against the PRDM16 protein sequence using the following search parameters: 10 ppm mass tolerance for precursor ions; 1 Da mass tolerance for product ions. The modification of 114.0429 mass units to lysine was included in the database searches to determine ubiquitin-modified peptides. All databases include a reversed version of all of the sequences, and the data were filtered to 1% or lower peptide FDR. The tandem MS data of matched ubiquitinated peptides were checked manually for their validity.

### Immunoprecipitation and immunoblot analyses

Two-step immunoprecipitation and ubiquitination assays were performed as described previously^39^. In the first-round immunoprecipitation assays, cell lysates were prepared using lysis buffer (50 mM Tris-Cl pH 7.4, 150 mM NaCl, 1% Triton X-100 and 1 mM EDTA) supplemented with a protease inhibitor cocktail (Roche), and mixed with the anti-Flag M2 affinity gel for 2 h. The agarose gels were washed extensively using the same buffer. In the second-round immunoprecipitation assays, the bound proteins on the agarose were denatured by boiling for 5 min in the lysis buffer containing 1% SDS. The solution was diluted 1:10 using lysis buffer. The diluted elutes were re-immunoprecipitated with the anti-Flag M2 affinity gel. After four washes, the bound proteins were separated by SDS–PAGE and analysed using immunoblotting.

### Cycloheximide assay

A published protocol was used in our study^40^. In brief, differentiated adipocytes or HEK293T cells transfected with the indicated plasmids were treated with cycloheximide at a final concentration of 20 µg ml^−1^ and 10 µg ml^−1^, respectively. The cells were collected at the indicated time points. Cell lysates were analysed by immunoblotting for PRDM16. The intensity of PRDM16-specific protein expression was quantified by Image J software and normalized to that of β-actin signals. For the statistical analyses, two-tailed unpaired Student’s *t*-tests were performed on the basis of *n* = 3 biologically independent samples.

### GST pull-down

Various forms of GST-tagged PRDM16 protein were expressed in *E. Coli* and purified as previously described^4^. The GST–PRDM16 protein fragments were incubated with pre-equilibrated Glutathione-Sepharose beads (GE Healthcare) for 2 h, followed by extensive washing. The preloaded GST resins were incubated with Myc-tagged APPBP2 protein for 2 h at 4 °C. Precipitates were washed four times and separated by SDS–PAGE and then analysed using immunoblotting.

### OCR assays

OCR was measured using the Seahorse XFe Extracellular Flux Analyzer (Agilent) in a 24-well plate or 96-well plate. Differentiated inguinal-WAT-derived adipocytes were seeded and differentiated for 4 or 5 days. For the measurement of noradrenaline-induced respiration, differentiated adipocytes were stimulated with 1 µM noradrenaline. For the measurement of uncoupled respiration in a 24-well plate, cells were treated with 5 µM oligomycin, followed by phenylhydrazone (5 µM) and antimycin (5 µM). For the measurement of uncoupled respiration in a 96-well plate, cells were treated with 1 µM oligomycin, followed by phenylhydrazone (3 µM) and antimycin (0.5 µM). For the measurement of noradrenaline-induced tissue OCR, adipose tissues (0.5 mg for BAT, 1.5 mg for inguinal and 2.5 mg for epididymal WAT) were placed into XF24 Islet Capture Microplates and stimulated with 10 µM noradrenaline.

### RNA-seq and analyses

For RNA-seq analysis of inguinal cells with stable *Cul2* knockdown or scramble control, total RNA was isolated using the RNeasy Micro Kit (Qiagen). High-throughput sequencing was performed using the HiSeq 3000 instrument (Illumina) at the Technology Center for Genomics & Bioinformatics at UCLA. The reads were mapped to the latest UCSC transcript set using Bowtie2 v.2.1.0 and the gene expression level was estimated using RSEM (v.1.2.15)^41^. Trimmed mean of *M*-values (from edgeR) were used to normalize the gene expression. Gene Ontology analysis was performed using Enrichr^42^. RNA-seq and library construction were conducted by technical staff at the UCLA genome core who were blinded to the experimental groups. For RNA-seq analysis of *Prdm16*-KO mouse-derived inguinal adipocytes, total RNA was isolated using the Zymo Direct-zol RNA preparation kit (R2052, Zymo). Extracted RNA (400 ng) was treated with the NEBNext rRNA Depletion Kit v2 (E7400X) to deplete ribosomal RNA and then converted into double-stranded cDNA using the NEBNext mRNA Second Strand Synthesis Module (E6111L). cDNA was analysed using Qubit and BioAnalyzer and subsequently amplified for 12 cycles using the Nextera XT DNA Library Preparation Kit (Illumina FC-131). Generated libraries were analysed by Qubit and Agilent Bioanalyzer, pooled at a final concentration of 1.35 pM, and sequenced on the NextSeq 500 system. Sequencing reads were demultiplexed and trimmed for adapters using bcl2fastq (v.2.20.0). Secondary adapter trimming, NextSeq/Poly(G) tail trimming and read filtering were performed using fastp (v.0.20.1); low-quality reads and reads shorter than 24 nucleotides after trimming were removed from the read pool. Salmon (v.1.4.0)^43^ was used to simultaneously map and quantify reads to transcripts in the GENCODE M24 genome annotation of GRCm38/mm10 mouse assembly. Salmon was run using full selective alignment, with sequence-specific and fragment GC-bias correction turned on (the --seqBias and --gcBias options, respectively). Transcript abundances were collated and summarized to gene abundances using the tximport package for R^44^. Normalization and differential expression analysis were performed using edgeR. For differential gene expression analysis, genes were considered to be significant if they passed an FDR cut-off of FDR ≤ 0.05. The heat map of the RNA-seq transcriptome was generated using MetaboAnalyst (v.5.0)^45^.

### ChIP assays

ChIP assays were performed according to the established commercial protocol using the Thermo Fisher Scientific Pierce Magnetic ChIP Kit (26157, Thermo Fisher Scientific). In brief, differentiated *Appbp2-*KO adipocytes and control adipocytes were fixed in 1% formaldehyde for 10 min by gently swirling the dish and quenched with 1× glycine for 5 min at room temperature. The samples were washed twice with ice-cold PBS supplement with Halt Cocktail, collected and then placed into membrane extraction buffer containing protease/phosphatase inhibitors. After centrifugation at 9,000*g* for 3 min, the supernatant was removed. The nuclei were digested with MNase (ChIP grade) in MNase Digestion Buffer Working Solution at 37 °C for 15 min, followed by sonication to break the nuclear membrane on ice. Digested chromatin was centrifuged at 9,000*g* for 5 min, and antibodies were added for overnight incubation at 4 °C on a rotating platform. ChIP grade protein A/G magnetic beads were added for 2 h at 4 °C. The samples were washed with the IP wash buffer provided with the kit. The samples were eluted with 1× IP elution buffer at 65 °C for 30 min by vigorous shaking. The samples were subsequently treated with proteinase K followed by column-purification to recover the DNA. Target enrichment was calculated as the percentage of input. The target loci of PRDM16 were chosen on the basis of the previous study that performed ChIP–seq of *PRDM16* in brown adipocytes^46^. A list of the primer sequences is provided in Supplementary Table 1.

### Human SNP analyses

The metabolic traits of the *APPBP2* genetic variant (rs34146848) were obtained from the FinnMetSeq exome sequence data^33^. The analysis also can be found in the type 2 diabetes knowledge portal (https://t2d.hugeamp.org/) and Pheweb (http://pheweb.sph.umich.edu/FinMetSeq/variant/17:58525018-C-T). Population frequencies of the *APPBP2* genetic variant (rs34146848) can be found in the gnomAD browser (https://gnomad.broadinstitute.org/variant/17-58525018-C-T?dataset=gnomad_r2_1)^34^ in which the SNP is more frequently found in African and African American individuals (17,099 out of 41,342 alleles, for a frequency of 41.4%) compared with in other ethnic groups. Accordingly, we independently tested for the association between genetic variants at the *APPBP2* gene locus and measures of obesity (body mass index, waist–ratio and waist–hip ratio adjusted for BMI) in individuals of African ancestry (*n* = 7,447) in the UK Biobank^35^ using the BOLT linear mixed model GWAS software. Phenotypes were adjusted for age, age squared, sex, genotyping array and genetic principal components, followed by inverse normal transformation.

### Glucose homeostasis in mice

Male Adipo-*Cul2*-KO, Adipo-*Appbp2*-KO and the respective littermate control mice in the C57BL/6J background at 6 weeks old were fed on an HFD (60% fat, D12492, Research Diets) at 22 °C. Body weight was measured every week. The fat mass and lean mass of mice were measured on an HFD for 8 weeks using the Body Composition Analyzer EchoMRI (Echo Medical Systems) system. For glucose-tolerance tests, mice on an HFD for 3 weeks or 9 weeks and fasted for 6 h from 09:00 to 15:00 were administered glucose intraperitoneally (1.5 g kg^−1^ body weight). For the insulin-tolerance tests, mice on an HFD for 10 weeks and fasted for 3 h from 09:00 to 12:00 were injected intraperitoneally with insulin (1 U kg^−1^ body weight). For the pyruvate-tolerance tests, mice on an HFD for 11 weeks and fasted for 16 h were injected intraperitoneally with pyruvate (1 g kg^−1^ body-weight). Blood samples were collected at the indicated time points before and after injection, and glucose levels were measured using blood glucose test strips (Freestyle Lite).

### Energy expenditure in mice

Whole-body energy expenditure (VO_2_, VCO_2_), food intake and locomotor activity (beam break counts) of Adipo-*Cul2*-KO mice and littermate control mice were monitored using the Comprehensive Laboratory Animal Monitoring System (CLAMS, Columbus Instruments) after 3 weeks of HFD. For the analyses of Adipo-*Appbp2*-KO mice, the whole-body metabolic rate was measured using the Promethion Metabolic Cage System (Sable Systems) at 30 °C. Adipo-*Appbp2*-KO and their littermate control mice were acclimatized to 30 °C for 3 days before transferring to metabolic cages. During the measurement of energy expenditure, mice received a single intraperitoneal injection of CL-316,243 (Sigma-Aldrich; 0.1 mg per kg body weight). Obtained indirect calorimetry data were analysed by CaIR-ANCOVA (https://calrapp.org/), a regression-based analysis of energy expenditure in mice^47^.

### Fatty acid oxidation assay

Fatty acid oxidation assays were performed according to the protocol described by our previous work^48^. In brief, BAT, inguinal WAT and gastrocnemius muscle tissues were isolated from Adipo-*Cul2*-KO and control mice after exposure to 8 °C, or Adipo-*Appbp2*-KO on an HFD. The tissues were minced to small pieces, placed into a polypropylene round-bottom tube and then incubated in the 1 ml KRB-HEPES buffer containing 0.5 μCi ml^−1^ [1-^14^C]oleic acid at 37 °C at 60 rpm for 1 h. After adding 350 μl 30% hydrogen peroxide into the reaction mixture, [^14^C]CO_2_ was trapped in the centre well supplemented with 300 μl of 1 M benzethonium hydroxide solution for 20 min at room temperature. ^14^C radioactivity was measured using a liquid scintillation counter and normalized to tissue mass.

### Lipid profiling

For the measurement of liver triglyceride contents, liver tissues from Adipo-*Cul2-*KO or Adipo-*Appbp2-*KO mice were collected and homogenized in 350 ml ethanolic KOH (100% ethanol and 30% KOH at a ratio of 2:1) and incubated overnight at 55 °C. Subsequently, tissue lysates were supplemented with 50% ethanol to 1 ml final volume. After centrifugation, the supernatant was mixed with 1 M MgCl_2_ and incubated on ice for 10 min. The amounts of triglycerides were measured using the Infinity Triglycerides kit (Thermo Fisher Scientific). Serum cholesterol and serum triglyceride measurement were performed by the Longwood Small Animal Imaging Facility at BIDMC.

### Peripheral insulin signalling in vivo

Adipo-*Appbp2-*KO mice and their littermate controls at 4 weeks of HFD were fasted for 4 h followed by intraperitoneal injection of insulin at 1.3 U kg^−1^ body weight. Liver, EpiWAT and inguinal WAT were removed 10 min after the injection and lysed in RIPA lysis buffer, supplemented with protease and phosphatase inhibitor cocktails. The lysates were separated by SDS–PAGE and analysed using immunoblotting. PhosphoPlus Akt (Ser473) Antibody Duet was used for western blot analysis.

### Cold-tolerance test

Adipo-*Cul2*-KO mice, Adipo-*Appbp2-*KO mice and their respective littermate control mice were kept on a regular chow diet. Mice were acclimatized to 30 °C for 11 days and subsequently exposed to 8 °C for 6 h. The rectal temperatures of mice were monitored every 1 h using the TH-5 thermometer (Physitemp).

### Tissue histology

Adipose tissues and liver were fixed in 4% paraformaldehyde overnight at 4 °C, followed by dehydration in 70% ethanol. After the dehydration procedure, tissues were embedded in paraffin and cut into sections at a thickness of 5 μm. The sections were processed for haematoxylin and eosin staining according to the standard protocol at the BIDMC pathology core. Images were acquired using the Revolve microscope (ECHO Laboratories).

### RT–qPCR

Total RNA was prepared from cells using TRIzol reagents (Invitrogen) according to the manufacturer’s instructions. Total RNA extracted from tissues was obtained using TRIzol reagents plus RNeasy Mini Kit (Qiagen). RNA samples were reverse-transcribed using the iScript cDNA Synthesis Kit (Bio-Rad Laboratories) according to the provided protocol. The quantifications of gene transcripts were performed by qPCR using the ABI ViiA 7 PCR or QS6 cycler (Applied Biosystems). 36B4 or TBP served as an internal control. A list of the PCR primers used to amplify the target genes is provided in Supplementary Table 1.

### Quantification of the mtDNA copy number

Total DNA was isolated from mature adipocytes with the SpeeDNA Isolation Kit (MB6918, ScienCell) according to the manufacturer’s instructions. DNA concentrations were measured using the Nanodrop 2000 (Thermo Fisher Scientific) and diluted to final concentrations of 20 ng ml^−1^ with double-distilled H_2_O. The mtDNA copy number was amplified using primers specific for the mitochondrial *Cox1*, *Cox2*, *Cox3*, *Atp6* and *Atp8* genes and normalized to genomic DNA by amplification of the β-globin gene. A list of the primer sequences is provided in Supplementary Table 1.

### Lipid staining by Oil Red O

Cells were washed once with PBS, fixed in 4% paraformaldehyde for 15 min and then stained with Oil-Red-O solution for 10–20 min at ambient temperature. Subsequently, cells were washed three times with PBS followed by imaging with a Revolve microscope (ECHO Laboratories).

### Electron microscopy

Immortalized inguinal preadipocytes were differentiated for 5 days cultured on 12-well plates. Cells were fixed for 2 h at room temperature with fixative solution (2.5% glutaraldehyde, 1.25% paraformaldehyde, 0.03% picric acid in 0.1 M sodium cacodylate buffer, pH 7.4), washed in 0.1 M cacodylate buffer and post-fixed with 1% osmiumtetroxide (OsO_4_)/1.5% potassium ferrocyanide (KFeCN_6_) for 1 h. Samples were washed in water twice, 1× maleate buffer (MB) one time, and incubated in 1% uranyl acetate in MB for 1 h followed by two washes in water and subsequent dehydration in grades of alcohol (10 min each at 50%, 70% and 90%, and twice for 10 min at 100%). After dehydration, propyleneoxide was added to the dish and the cells were lifted off using a transfer pipet, pelleted and infiltrated overnight in a 1:1 mixture of propyleneoxide and TAAB Epon (TAAB Laboratories Equipment; https://taab.co.uk). The samples were then embedded in TAAB Epon and polymerized at 60 °C for 48 h. Ultrathin sections (about 60 nm) were cut on the Reichert Ultracut-S microtome, picked up onto copper grids stained with lead citrate and examined on the JEOL 1200EX transmission electron microscope or the TecnaiG^2^ Spirit BioTWIN system and images were recorded with the AMT 2k CCD camera.

### Statistics and reproducibility

All the biological experiments were repeated at least twice and reproduced. RNA-seq was performed once but three independent samples were analysed and further validated using alternative approaches, such as RT–qPCR. Western blotting data were confirmed by two or three independent samples. The presented data were collected from biologically independent samples. Statistical analyses were performed using GraphPad Prism v.7.0 (GraphPad). All data are represented as mean ± s.e.m. unless otherwise specified. Unpaired Student’s *t*-tests were used for two-group comparisons. One-way ANOVA followed by the Dunnett’s test was used for multiple-group comparisons. Two-way ANOVA was used for Seahorse measurements from multiple groups. Two-way repeated-measures ANOVA followed by Fisher’s LSD test was applied to determine the statistical differences in body-weight gain, whole-body energy expenditure results, glucose-tolerance tests, insulin-tolerance tests and pyruvate-tolerance tests between genotypes. The statistical parameters and mouse numbers used per experiment are specified in the figure legends. No statistical methods were used to predetermine sample size. *P* < 0.05 was considered to be significant throughout the study.

### Reporting summary

Further information on research design is available in the Nature Research Reporting Summary linked to this article.

## Online content

Any methods, additional references, Nature Research reporting summaries, source data, extended data, supplementary information, acknowledgements, peer review information; details of author contributions and competing interests; and statements of data and code availability are available at 10.1038/s41586-022-05067-4.

## Supplementary information

Supplementary Fig. 1 Uncropped gels and western blots for data shown in the Figures and Extended Data Figures.

Reporting Summary

Supplementary Table 1 Sequences of primers used for mouse genotyping, gRNA, shRNA, RT–qPCR and ChIP–qPCR.

Supplementary Table 2 Exact *P* values that are not displayed in some of the figures.

## Data Availability

RNA-seq reads used in Fig. 1d and Extended Data Fig. 2i are available at the NCBI Sequence Read Archive repository under accession number PRJNA758917. The RNA-seq data relating to Fig. 4c have been deposited at the NCBI Gene Expression Omnibus under accession number GSE196699. All unique materials used are available from the authors on request. Other materials are available from commercial sources as described in the text. Source data are provided with this paper.

## Source data

Source Data Fig. 1
Source Data Fig. 2
Source Data Fig. 3
Source Data Fig. 4
Source Data Fig. 5
Source Data Extended Data Fig. 1
Source Data Extended Data Fig. 2
Source Data Extended Data Fig. 3
Source Data Extended Data Fig. 4
Source Data Extended Data Fig. 5
Source Data Extended Data Fig. 6
Source Data Extended Data Fig. 7
Source Data Extended Data Fig. 8
Source Data Extended Data Fig. 9
Source Data Extended Data Fig. 11
Source Data Extended Data Fig. 12
Source Data Extended Data Fig. 13
