# Supplementary material for: Post-translational control of beige fat biogenesis by PRDM16 stabilization
Source: Nature. 2022 Aug 17;609(7925):151–8. doi: 10.1038/s41586-022-05067-4 (PMC9433319; doi:10.1038/s41586-022-05067-4)

nature portfolio

https://doi.org/10.1038/s41586-022-05067-4

Supplementary information

Post-translational control of beige fat biogenesis by PRDM16 stabilization

In the format provided by the authors and unedited

Fig.1a

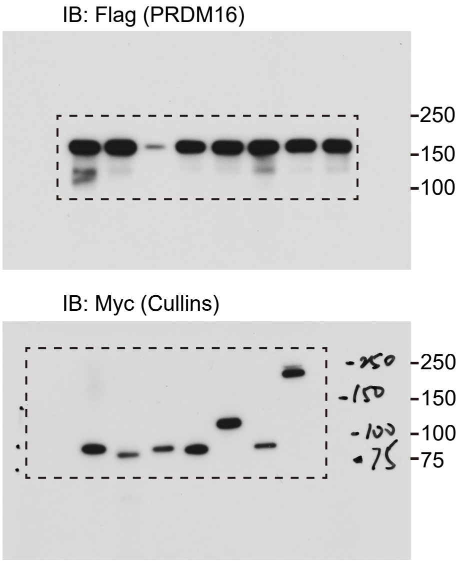

Fig.1b

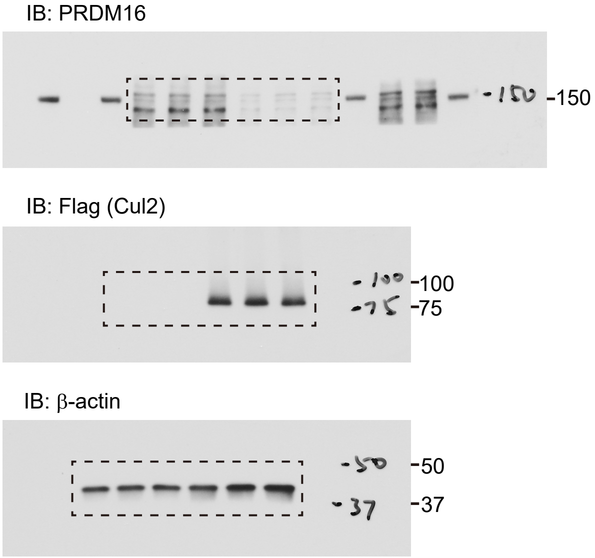

Fig.2a

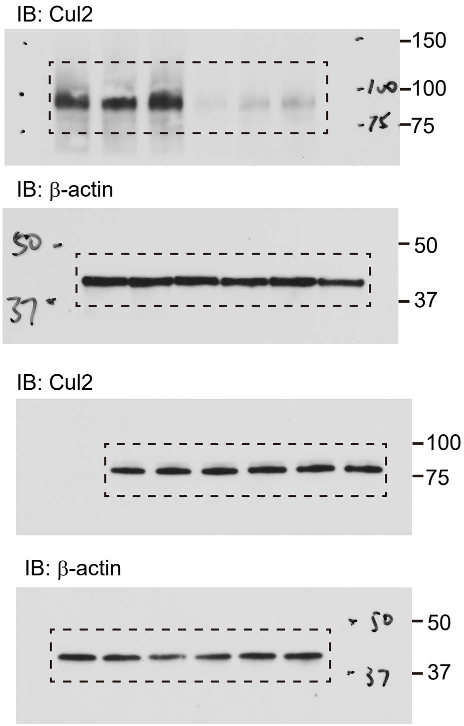

Fig.2d

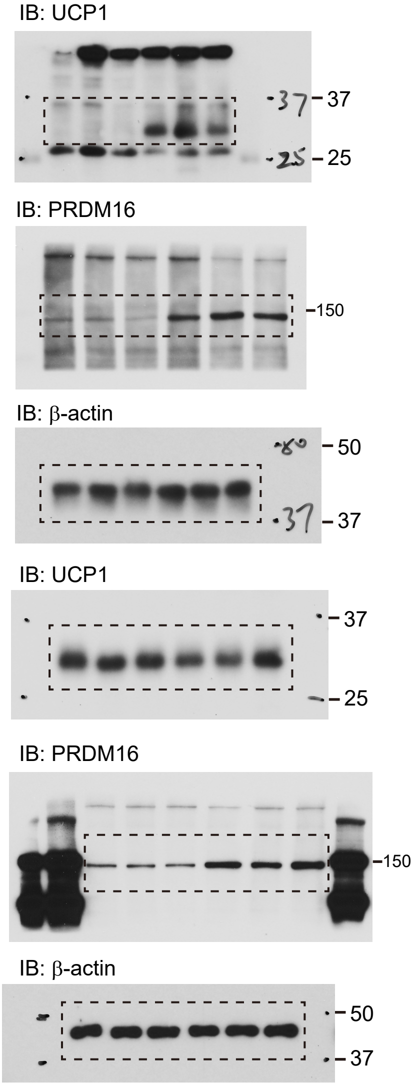

Fig.3d

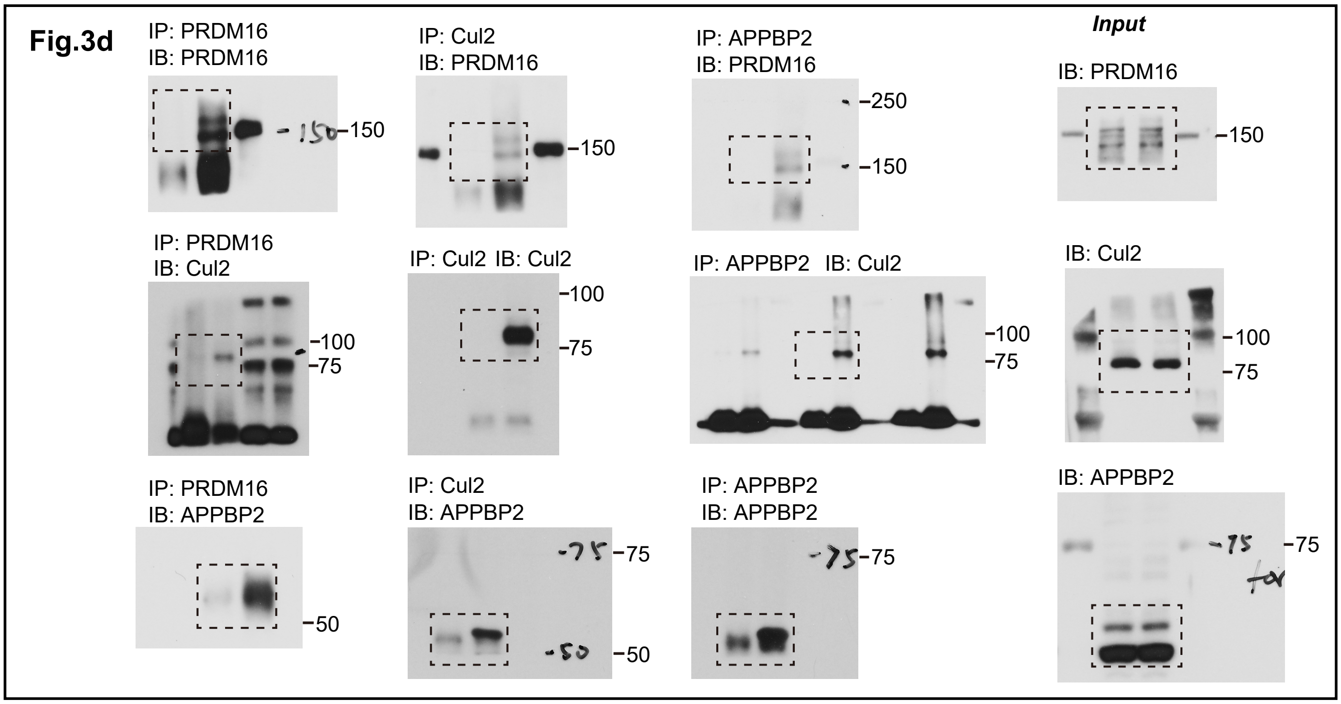

Fig.3e

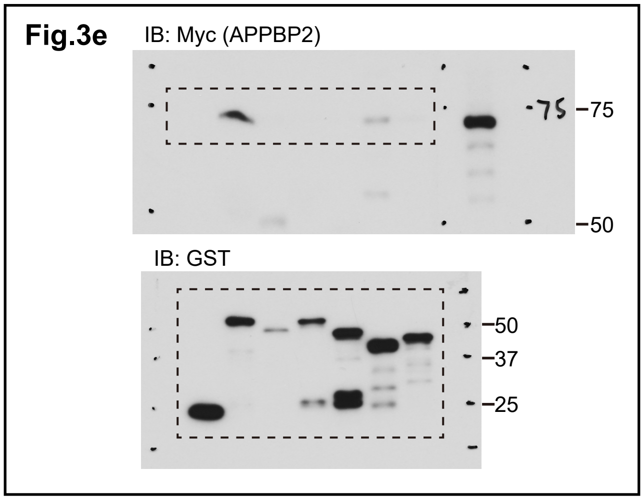

Fig.3f

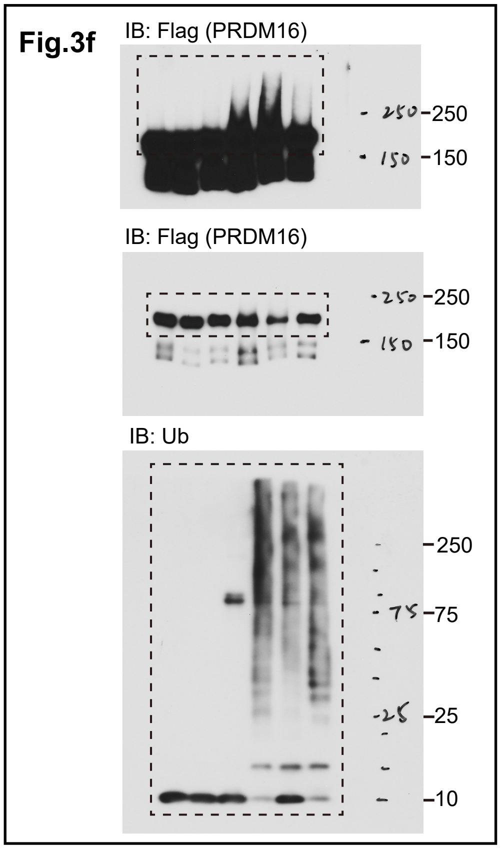

Fig.3h

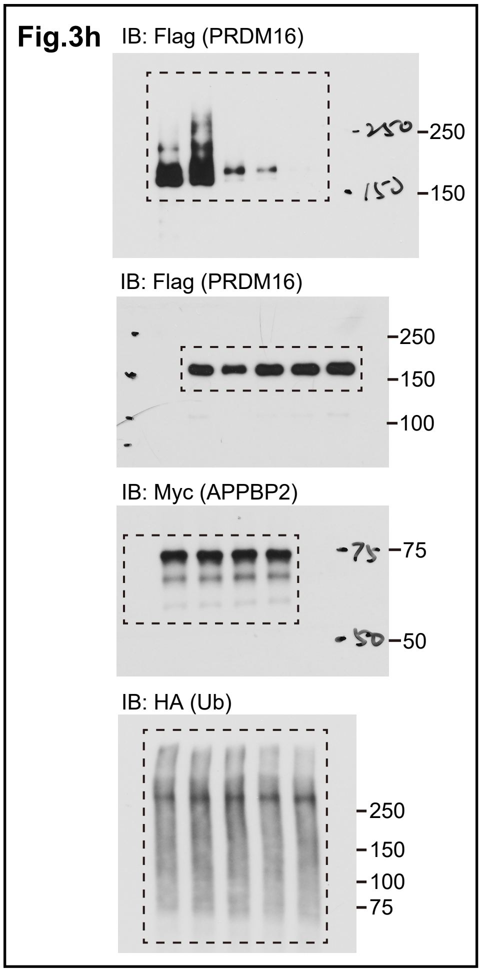

Fig.3i

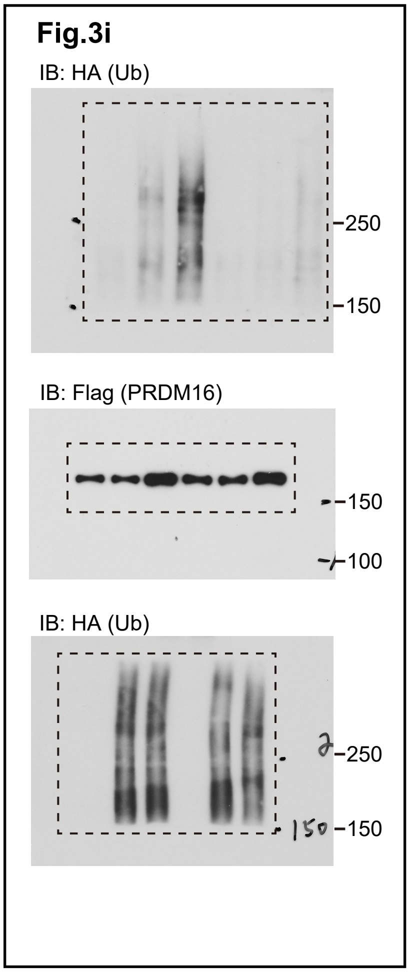

Fig.5a

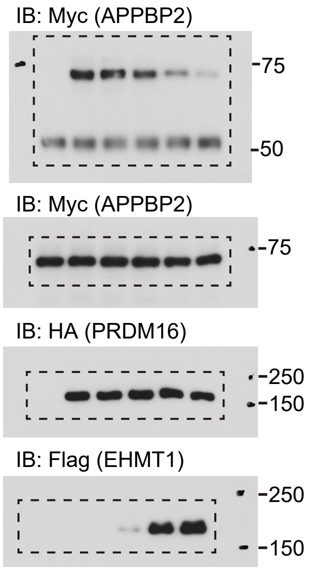

Fig.5b

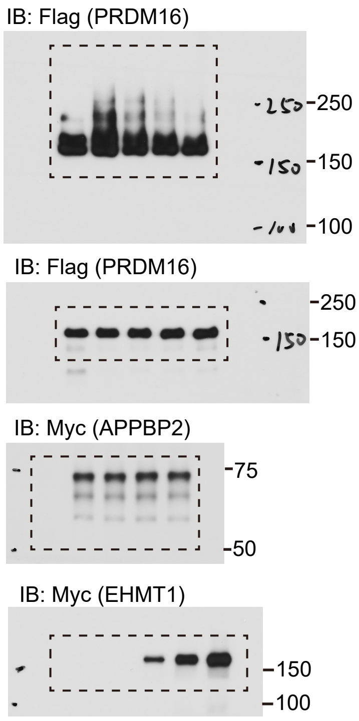

Fig.5c

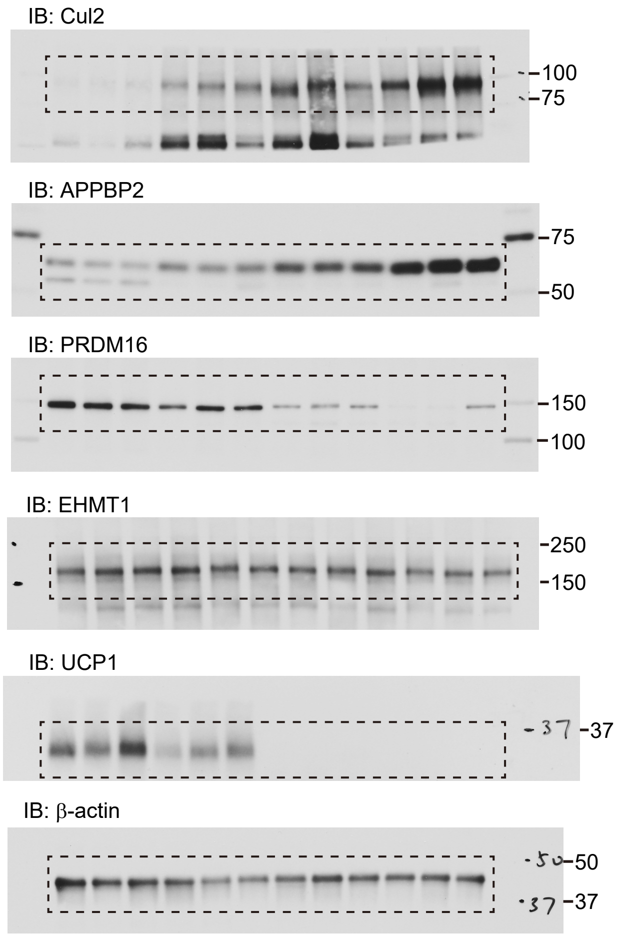

Extended Data Fig.1a

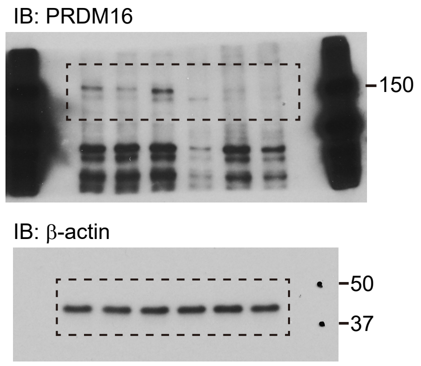

Extended Data Fig.1c

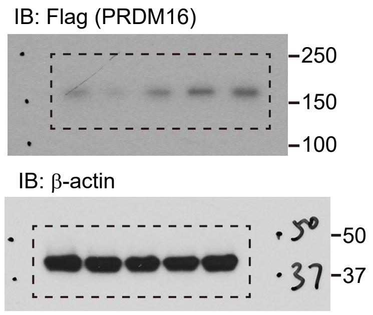

Extended Data Fig.1f

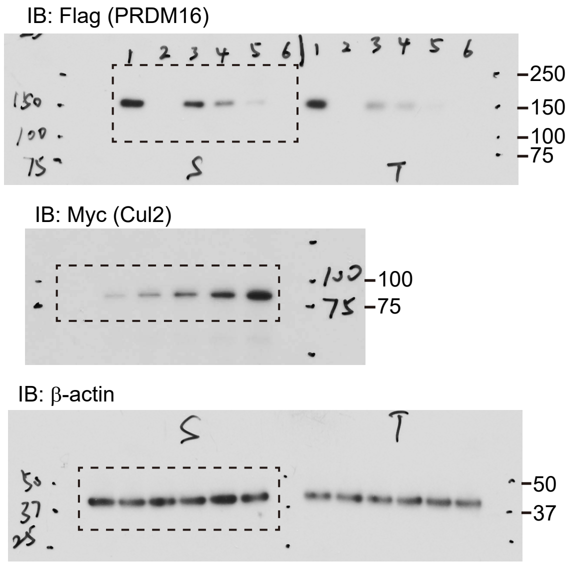

Extended Data Fig.1i

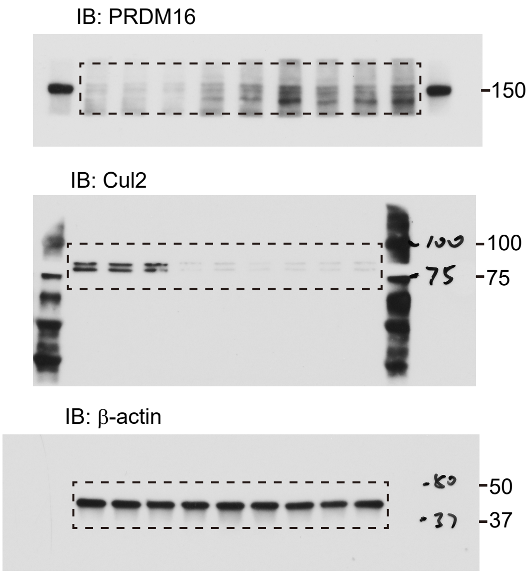

Extended Data Fig.1k

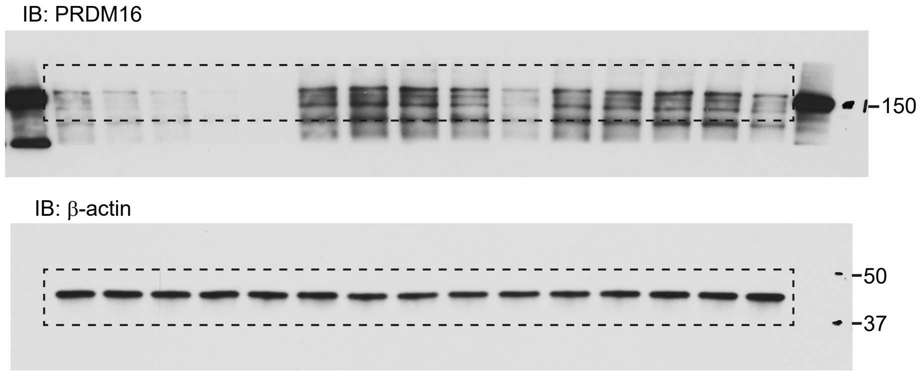

Extended Data Fig.2d

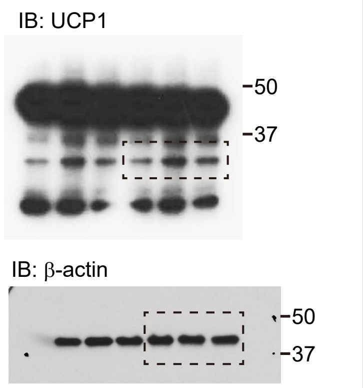

Extended Data Fig.3c

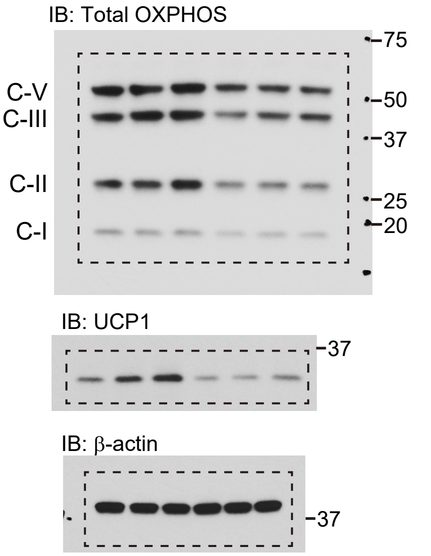

Extended Data Fig.4c

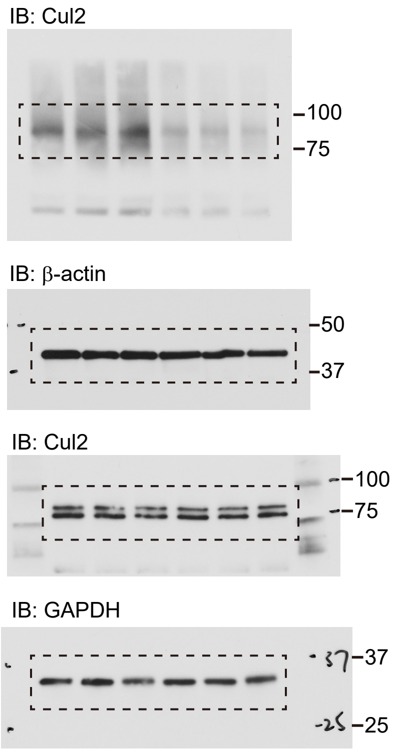

Extended Data Fig.3a

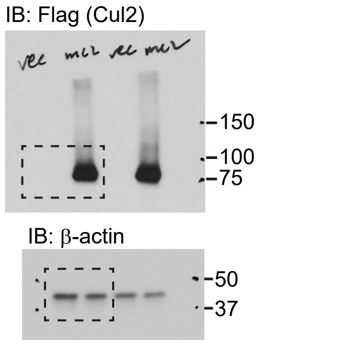

Extended Data Fig.6b

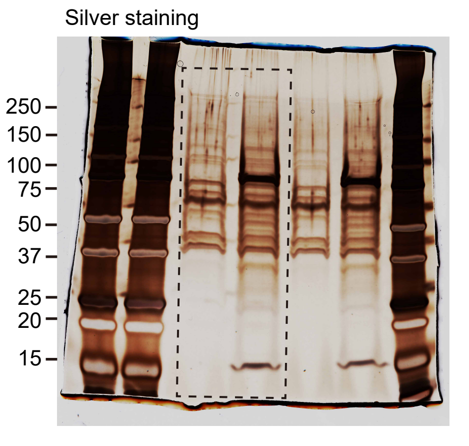

Extended Data Fig.6h

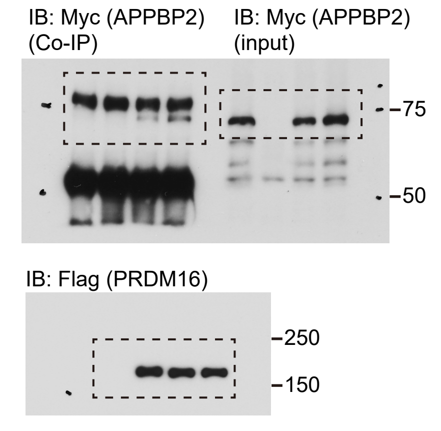

Extended Data Fig.6i

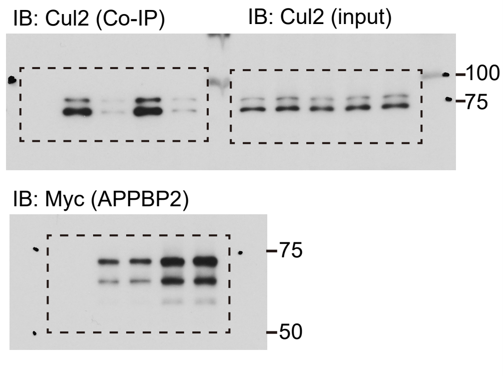

Extended Data Fig.7a

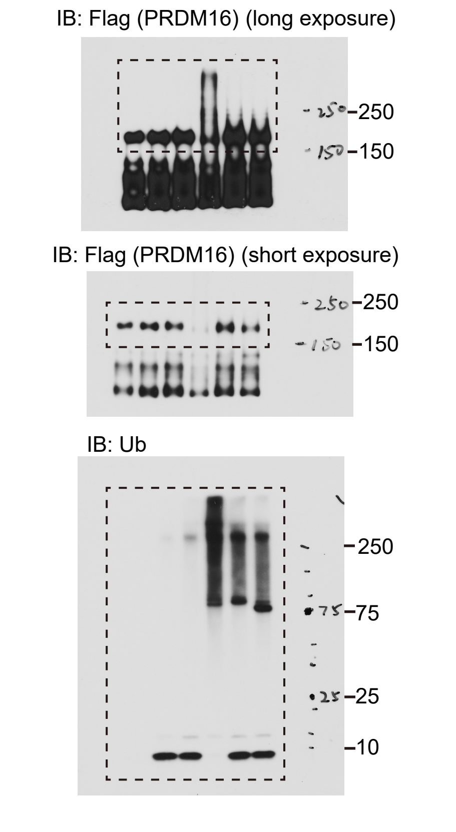

Extended Data Fig.7d

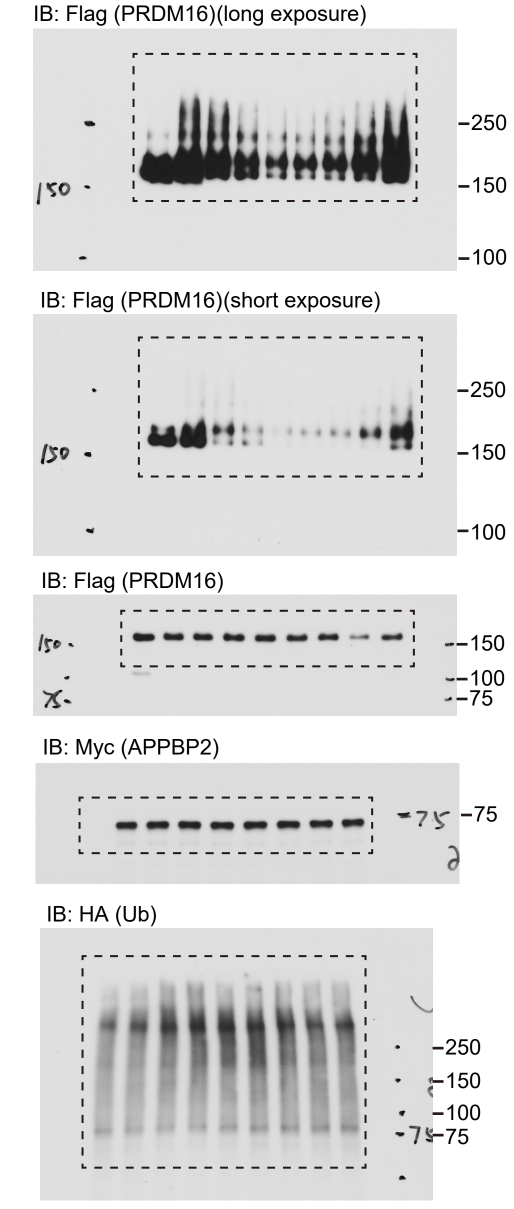

Extended Data Fig.7e

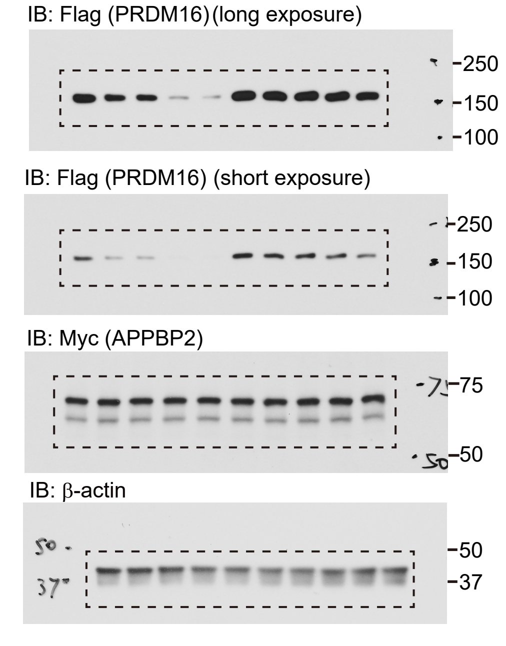

Extended Data Fig.8a

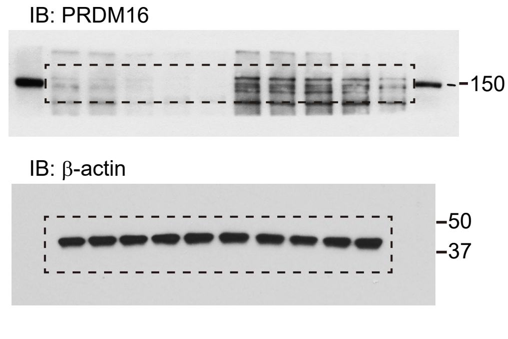

Extended Data Fig.7b

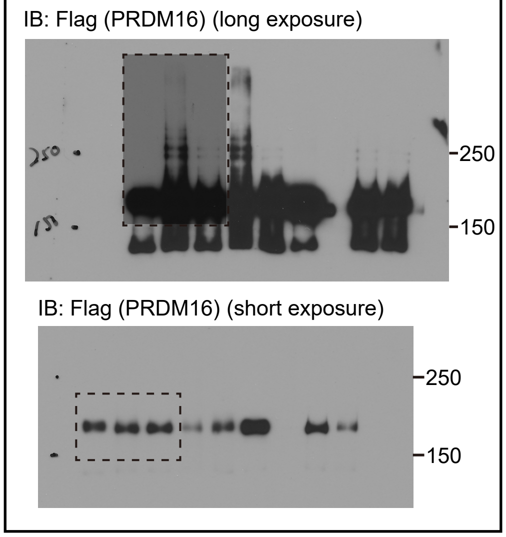

Extended Data Fig.8c

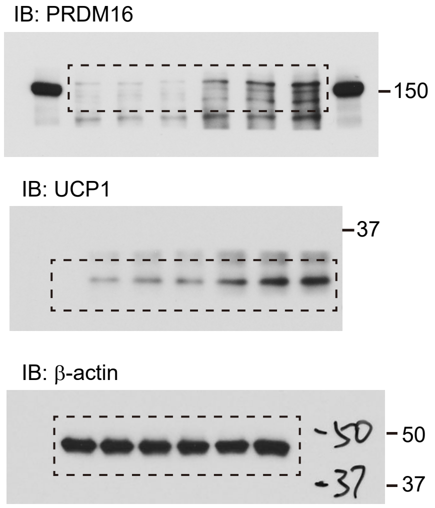

Extended Data Fig.9c

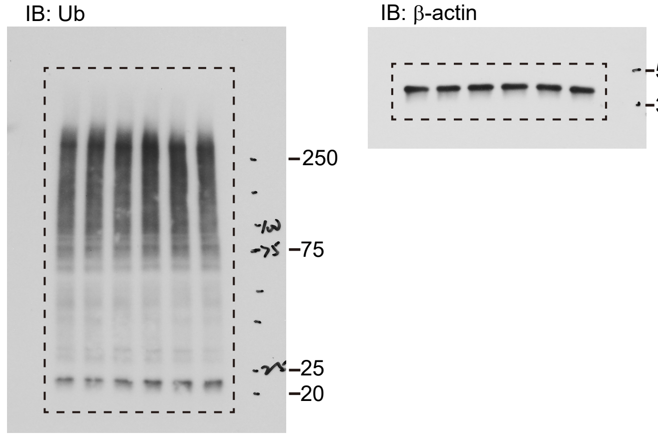

Extended Data Fig.9a

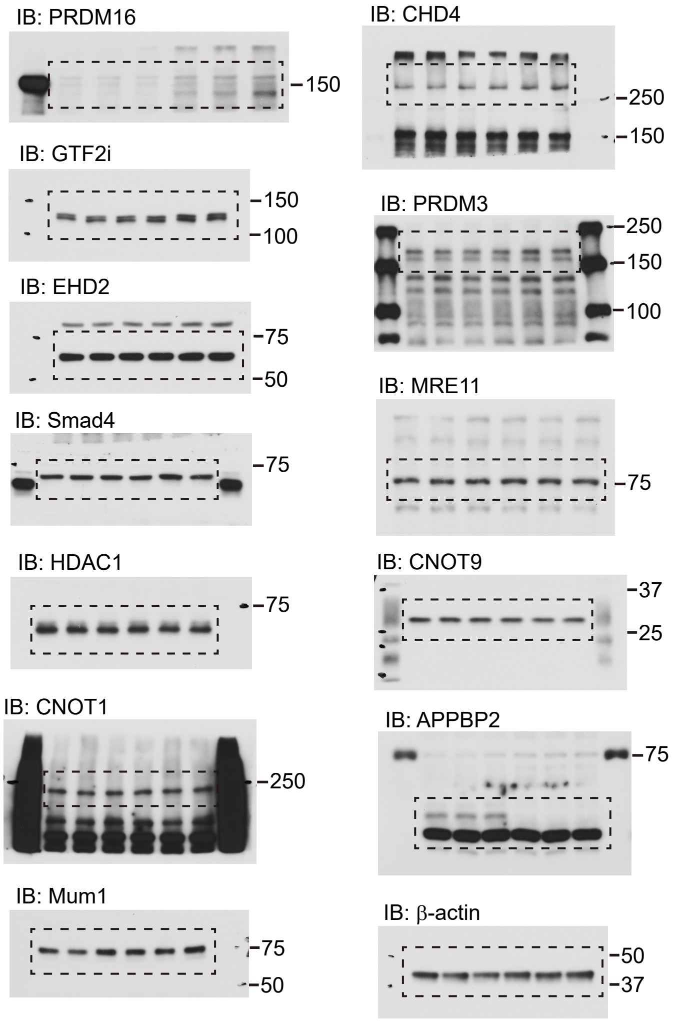

Extended Data Fig.9f

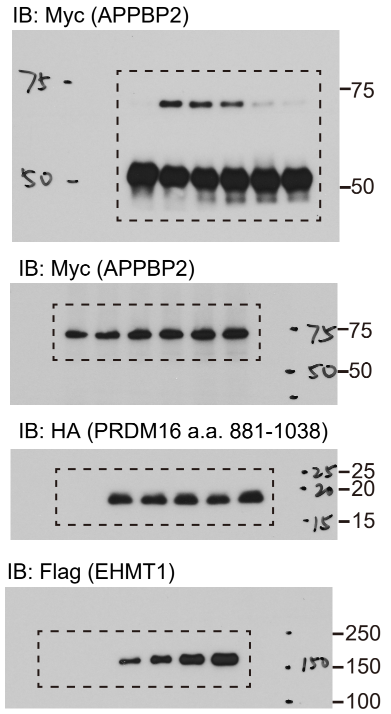

Extended Data Fig.9g

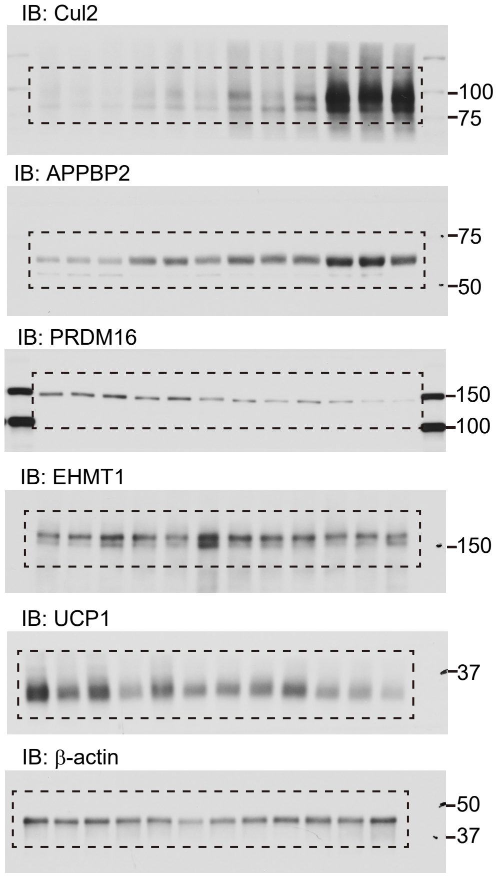

Extended data Fig.11a

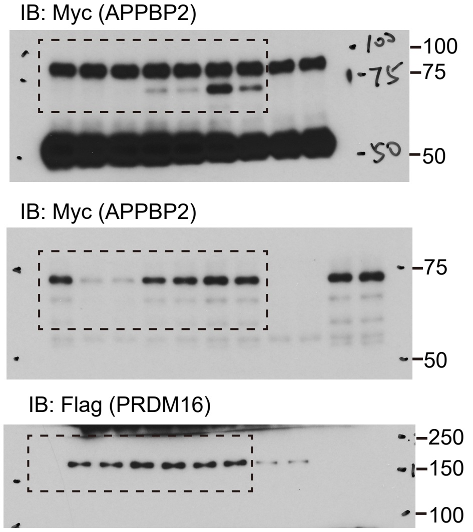

Extended data Fig.11b

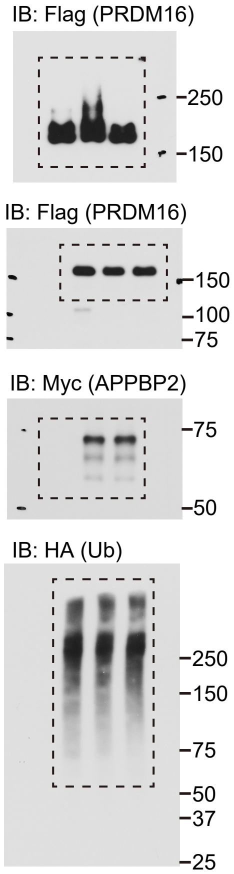

Extended data Fig.9h

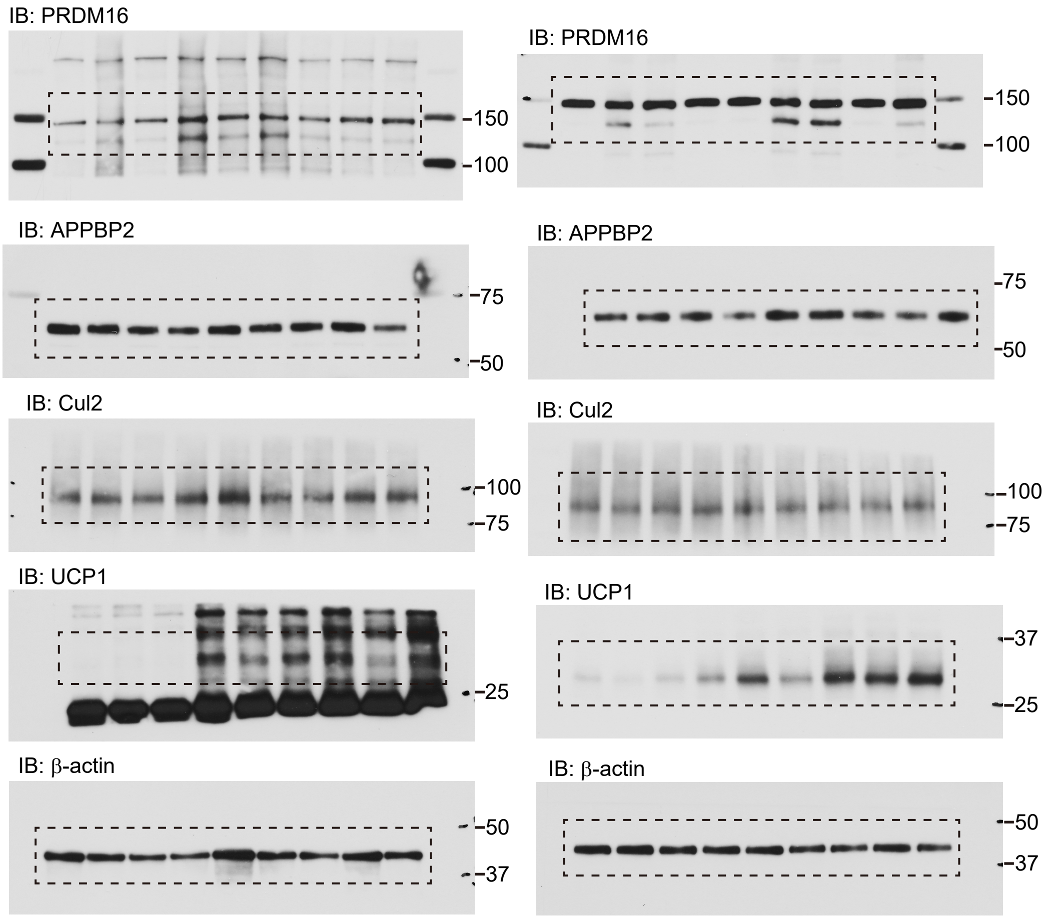

Extended data Fig.12b

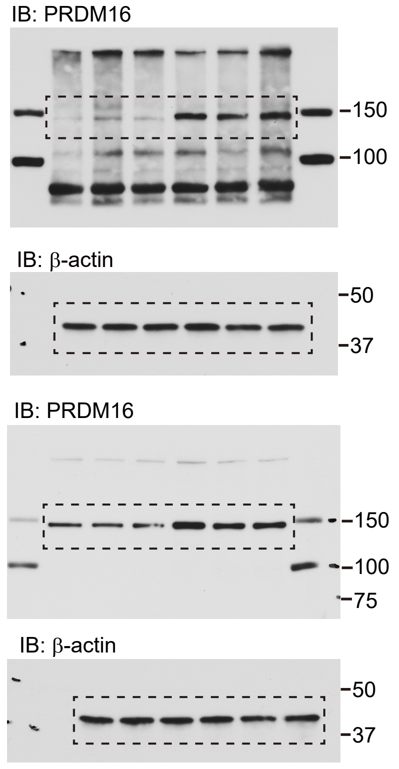

Extended data Fig.12k

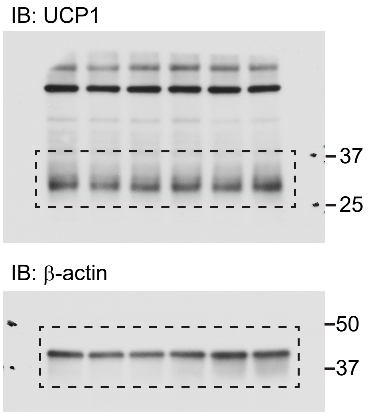

Extended data Fig.13k

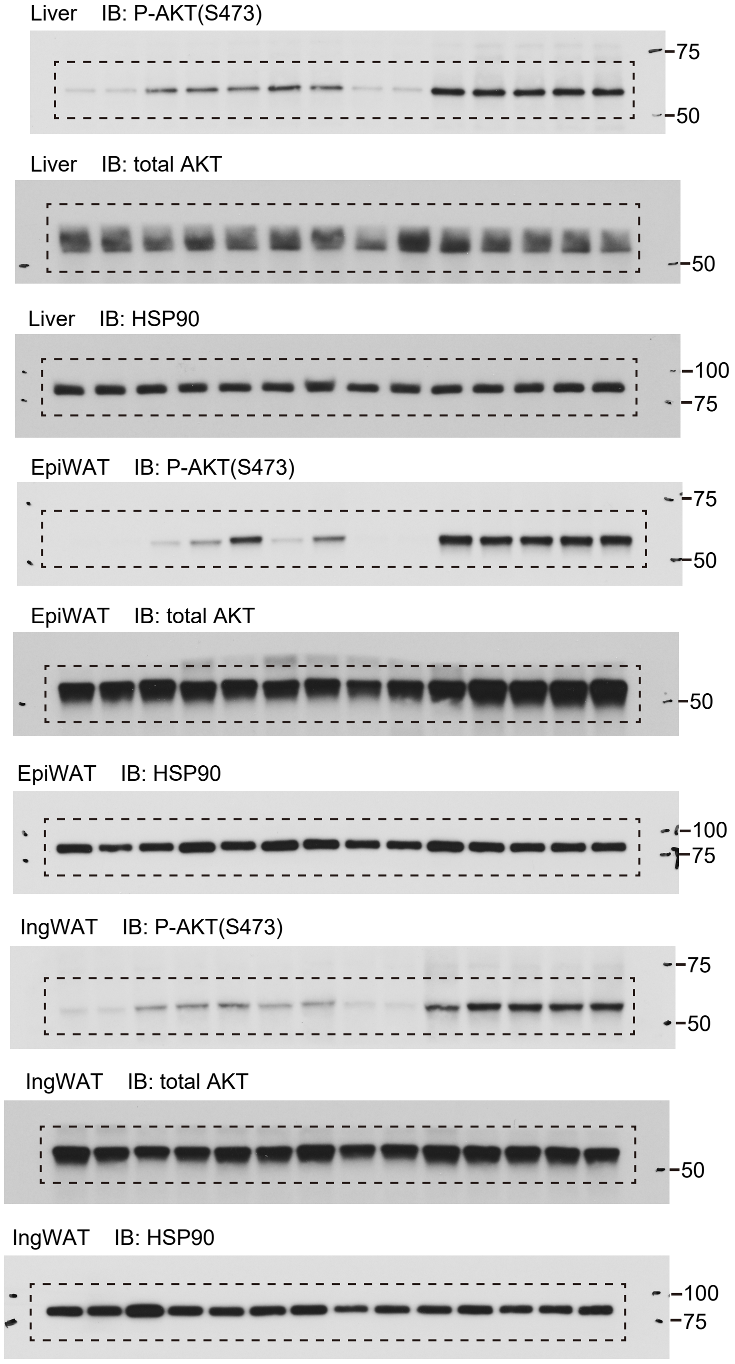

Supplement: Supplementary file 1 — Uncropped gels and western blots for data shown in the Figures and Extended Data Figures. [file 41586_2022_5067_MOESM1_ESM.pdf]
