# Supplementary material for: Post-translational control of beige fat biogenesis by PRDM16 stabilization
Source: Nature. 2022 Aug 17;609(7925):151–8. doi: 10.1038/s41586-022-05067-4 (PMC9433319; doi:10.1038/s41586-022-05067-4)
Supplement: Supplementary file 2 — Reporting Summary [file 41586_2022_5067_MOESM2_ESM.pdf]

nature portfolio

Corresponding author(s): Shingo Kajimura
Last updated by author(s): Jun 24, 2022

Reporting Summary

Nature Portfolio wishes to improve the reproducibility of the work that we publish. This form provides structure for consistency and transparency in reporting. For further information on Nature Portfolio policies, see our Editorial Policies and the Editorial Policy Checklist.

Statistics

For all statistical analyses, confirm that the following items are present in the figure legend, table legend, main text, or Methods section.

n/a	Confirmed	
☐	☒	The exact sample size (*n*) for each experimental group/condition, given as a discrete number and unit of measurement
☐	☒	A statement on whether measurements were taken from distinct samples or whether the same sample was measured repeatedly
☐	☒	The statistical test(s) used AND whether they are one- or two-sided<br>*Only common tests should be described solely by name; describe more complex techniques in the Methods section.*
☒	☐	A description of all covariates tested
☐	☒	A description of any assumptions or corrections, such as tests of normality and adjustment for multiple comparisons
☐	☒	A full description of the statistical parameters including central tendency (e.g. means) or other basic estimates (e.g. regression coefficient) AND variation (e.g. standard deviation) or associated estimates of uncertainty (e.g. confidence intervals)
☐	☒	For null hypothesis testing, the test statistic (e.g. *F*, *t*, *r*) with confidence intervals, effect sizes, degrees of freedom and *P* value noted<br>*Give P values as exact values whenever suitable.*
☒	☐	For Bayesian analysis, information on the choice of priors and Markov chain Monte Carlo settings
☒	☐	For hierarchical and complex designs, identification of the appropriate level for tests and full reporting of outcomes
☒	☐	Estimates of effect sizes (e.g. Cohen's *d*, Pearson's *r*), indicating how they were calculated

Our web collection on statistics for biologists contains articles on many of the points above.

Software and code

Policy information about availability of computer code

Data collection	Comprehensive Laboratory Animal Monitoring System (CLAMS, Columbus Instruments)<br>Promethion Metabolic Cage System (Sable Systems)<br>Body Composition Analyzer EchoMRI (Echo Medical Systems)<br>core-body temperature: TH-5 thermometer (Physitemp)<br>RNAseq analysis: Bowtie2 version 2.1.0; RSEM v1.2.15; Enrichr; bcl2fastq (v2.20.0); fastp (v0.20.1); Salmon (v1.4.0); tximport package for R; edgeR; MetaboAnalyst 5.0<br>QPCR: QuantStudio Real-time PCR system 1.2v<br>Seahorse: Wave 2.6<br>Electron microscopy: JEOL 1200EX Transmission electron microscope or a TecnaiG² Spirit BioTWIN; AMT 2k CCD camera
Data analysis	GraphPad Prism 7.0 (GraphPad Software); CalR version 1.3; Image J software 1.52a

For manuscripts utilizing custom algorithms or software that are central to the research but not yet described in published literature, software must be made available to editors and reviewers. We strongly encourage code deposition in a community repository (e.g. GitHub). See the Nature Portfolio guidelines for submitting code & software for further information.

Data

Policy information about availability of data

All manuscripts must include a data availability statement. This statement should provide the following information, where applicable:

- Accession codes, unique identifiers, or web links for publicly available datasets
- A description of any restrictions on data availability
- For clinical datasets or third party data, please ensure that the statement adheres to our policy

RNA-sequencing reads used in Fig. 1d and Extended data Fig. 2i are available in the Sequence Read Archive (SRA) repository at NCBI under the accession number PRJNA758917. The RNA-seq data related to Fig. 4c have been deposited in NCBI's Gene Expression Omnibus and are accessible through GEO Series accession number GSE196699.

Field-specific reporting

Please select the one below that is the best fit for your research. If you are not sure, read the appropriate sections before making your selection.

☒ Life sciences ☐ Behavioural & social sciences ☐ Ecological, evolutionary & environmental sciences

For a reference copy of the document with all sections, see nature.com/documents/nr-reporting-summary-flat.pdf

Life sciences study design

All studies must disclose on these points even when the disclosure is negative.

Sample size	No statistical methods were used to predetermine sample size. The sample size for our animal experiments in this study was based on our experience with experimental models, anticipated biological variables, and previous literatures. Sample numbers were described in the Figure legends.
Data exclusions	No data were excluded in the study.
Replication	All the biological experiments were repeated, at least, twice and reproduced. RNA-sequencing were performed once but three independent samples were analyzed and further validated by alternative approaches, such as qRT-PCR. Western blotting data were confirmed by two or three independent samples. At least 3 independent mice cohorts (Adipo-Cul2 and Adipo-Appbp2, respectively) on the high-fat diet study were performed. The result of a cohort of n=15 for littermate controls and n=12 for Adipo-Cul2KO mice was presented in Fig. 2. The result of a cohort of n=11 for littermate controls and n=10 for Adipo-Appbp2 KO mice was presented in Fig. 5.
Randomization	Mice were randomly assigned at the time of purchase or weaning to minimize any potential bias.
Blinding	RNA sequencing and library constructions were performed by technical staffs at the UCLA genome core who were blinded to the experimental groups. RNA sequencing alignment and analyses were performed by the authors who were blinded to the experimental groups. Mice whole body metabolic rate using Comprehensive Laboratory Animal Monitoring System and the Promethion Metabolic Cage System were performed by technical staffs at UCSF or BIDMC who were blinded to the experimental groups. Blinding was not relevant to the other experiments in mice or cells because mice or cells had to be genotyped by PCR.

Reporting for specific materials, systems and methods

We require information from authors about some types of materials, experimental systems and methods used in many studies. Here, indicate whether each material, system or method listed is relevant to your study. If you are not sure if a list item applies to your research, read the appropriate section before selecting a response.

Materials & experimental systems

n/a	Involved in the study	
☐	☒	Antibodies
☐	☒	Eukaryotic cell lines
☒	☐	Palaeontology and archaeology
☐	☒	Animals and other organisms
☒	☐	Human research participants
☒	☐	Clinical data
☒	☐	Dual use research of concern

Methods

n/a	Involved in the study	
☒	☐	ChIP-seq
☒	☐	Flow cytometry
☒	☐	MRI-based neuroimaging

Antibodies

Antibodies used	The following antibodies were used in this study: UCP1 antibody (ab-10983, Abcam; 1:1000 dilution in immunoblotting), UCP1

Antibodies used	antibody (U6382, Sigma; 1:1000 dilution in immunoblotting), Cul2 antibody (sc-166506, Santa Cruz; 1:2000 dilution in immunoblotting; 1:50 dilution in immunoprecipitation), Recombinant CUL-2 antibody [EPR3104(2)] (ab166917, Abcam; 1:2000 dilution in immunoblotting; 1:50 dilution in immunoprecipitation), APPBP2 antibody (NBP2-81781, Novus; 1:1000 dilution in immunoblotting; 1:50 dilution in immunoprecipitation), Flag-HRP antibody (A8592, Sigma; 1:3000 dilution in immunoblotting), HA antibody (sc-7392, Santa Cruz; 1:2000 dilution in immunoblotting), Myc antibody (sc-40, Santa Cruz; 1:2000 dilution in immunoblotting), PPARγ (E-8) (sc-7273, Santa Cruz; 1:50 dilution in ChIP), OXPHOS cocktail (ab110413, Abcam; 1:5000 dilution in immunoblotting), Ubiquitin antibody (sc-8017, Santa Cruz; 1:1000 dilution in immunoblotting), GST antibody (sc-138, Santa Cruz; 1:5000 dilution in immunoblotting), GAPDH antibody (sc-32233, Santa Cruz; 1:5000 dilution in immunoblotting), β-actin antibody (A3854, Sigma; 1:10000 dilution in immunoblotting), MUM1 (12682-1-AP, Proteintech; 1:1000 dilution in immunoblotting), SMAD4 (10231-1-AP, Proteintech; 1:1000 dilution in immunoblotting), EHD2 (11440-1-AP, Proteintech; 1:1000 dilution in immunoblotting), GTF2I (10499-1-AP, Proteintech; 1:1000 dilution in immunoblotting), HDAC1 (10197-1-AP, Proteintech; 1:1000 dilution in immunoblotting), CNOT1 (14276-1-AP, Proteintech; 1:1000 dilution in immunoblotting), CNOT9 (22503-1-AP, Proteintech; 1:1000 dilution in immunoblotting), CHD4 (14173-1-AP, Proteintech; 1:1000 dilution in immunoblotting), PRDM3 (C50E12) (2593S, Cell Signaling Technology; 1:1000 dilution in immunoblotting), EHMT1(E6Q8B) (35005S, Cell Signaling Technology; 1:1000 dilution in immunoblotting), PhosphoPlus® Akt (Ser473) Antibody Duet (8200S, Cell Signaling Technology; 1:2000 dilution in immunoblotting), HSP90 (4874S, Cell Signaling Technology; 1:2000 dilution in immunoblotting), normal mouse IgG (sc-2025, Santa Cruz; 1:100 dilution in immunoprecipitation), Rabbit IgG, polyclonal-Isotype Control (ab37415, Abcam; 1:1500 dilution in immunoprecipitation), Goat anti-Rabbit Light Chain Secondary Antibody (NBP2-75935, Novus; 1:5000 dilution in immunoblotting), Goat Anti-Mouse IgG, Light-Chain Specific Antibody (91196S, Cell Signaling Technology; 1:5000 dilution in immunoblotting), PRDM16 (AF6295, R&D systems; 1:50 dilution in immunoprecipitation), polyclonal antibody against PRDM16 generated by immunizing rabbit with recombinant human PRDM16 (GenScript, 1:2000 dilution in immunoblotting; 1:50 dilution in immunoprecipitation and ChIP).
Validation	Antibodies were validated based on the size of band in western blotting (molecular weight), specificity/selectivity assessed by using samples from knockout mouse/knockdown mouse/knockdown cells/over expression cells, and reproducibility of the results.<br>-Anti-Cul2 antibody: Fig. 1c, 2a; ED Fig. 4b<br>-Anti-APPBP2 antibody: Fig. 4f<br>-Anti-PRDM16 antibody: Fig. 1b, 1c, 2d, 4a, 4f, 5c; ED Fig. 1a, 1g, 7f, 8g, 8h<br>-Anti-UCP1 antibody: https://www.abcam.com/ucp1-antibody-ab10983.html; https://www.sigmaaldrich.com/US/en/product/sigma/u6382<br>-Anti-Cul2 antibody: https://www.scbt.com/p/cul-2-antibody-c-4?productCanUrl=cul-2-antibody-c-4&_requestid=1358332<br>-Anti-Recombinant CUL-2 antibody: https://www.abcam.com/cullin-2cul-2-antibody-epr31042-ab166917.html<br>-Anti-APPBP2 antibody: https://www.novusbio.com/products/appbp2-antibody_nbp2-81781<br>-Anti-Flag-HRP antibody: https://www.sigmaaldrich.com/US/en/product/sigma/a8592<br>-Anti-HA antibody: https://www.scbt.com/p/ha-probe-antibody-f-7?gclid=EAIaIQobChMIqea6ioP-9wIVRIVaBR3Ktg9pEAAYASAAEgJvqvD_BwE<br>-Anti-Myc antibody: https://www.scbt.com/p/c-myc-antibody-9e10?productCanUrl=c-myc-antibody-9e10&_requestid=1359589<br>-Anti-PPARγ: https://www.scbt.com/p/ppargamma-antibody-e-8?requestFrom=search<br>-Anti-OXPHOS cocktail: https://www.abcam.com/total-oxphos-rodent-wb-antibody-cocktail-ab110413.html<br>-Anti-Ubiquitin antibody: https://www.scbt.com/p/ubiquitin-antibody-p4d1?gclid=EAIaIQobChMIiK_ho4T-9wIVUyqzAB1eeAmXEAAYASAAEgJlj_D_BwE<br>-Anti-GST antibody: https://www.scbt.com/p/gst-antibody-b-14<br>-Anti-GAPDH antibody: https://www.scbt.com/p/gapdh-antibody-6c5?productCanUrl=gapdh-antibody-6c5&_requestid=1360829<br>-Anti-β-actin antibody: https://www.sigmaaldrich.com/US/en/product/sigma/a3854<br>-Anti-MUM1: https://www.ptgcn.com/products/MUM1-Antibody-12682-1-AP.htm<br>-Anti-SMAD4: https://www.ptgcn.com/products/SMAD4-Antibody-10231-1-AP.htm<br>-Anti-EHD2: https://www.ptgcn.com/products/EHD2-Antibody-11440-1-AP.htm<br>-Anti-GTF2I: https://www.ptgcn.com/products/GTF2I-Antibody-10499-1-AP.htm<br>-Anti-HDAC1: https://www.ptgcn.com/products/HDAC1-Antibody-10197-1-AP.htm<br>-Anti-CNOT1: https://www.ptgcn.com/products/CNOT1-Antibody-14276-1-AP.htm<br>-Anti-CNOT9: https://www.ptgcn.com/products/RQCD1-Antibody-22503-1-AP.htm<br>-Anti-CHD4: https://www.ptgcn.com/products/CHD4-Antibody-14173-1-AP.htm<br>-Anti-PRDM3: https://www.cellsignal.com/products/primary-antibodies/evi-1-c50e12-rabbit-mab/2593?site-search-type=Products&N=4294956287&Ntt=2593s&fromPage=plp&_requestid=1591500<br>-Anti-EHMT1: https://www.cellsignal.com/products/primary-antibodies/ehmt1-e6q8b-rabbit-mab/35005?site-search-type=Products&N=4294956287&Ntt=e6q8b%29+%2835005s&fromPage=plp&_requestid=1591652<br>-Anti-PhosphoPlus® Akt(Ser473) Antibody Duet: https://www.cellsignal.com/products/primary-antibodies/akt-ser473-antibody-duet/8200<br>-Anti-HSP90 antibody: https://www.cellsignal.com/products/primary-antibodies/hsp90-antibody/4874?site-search-type=Products&N=4294956287&Ntt=4874s&fromPage=plp&_requestid=1750770<br>-normal mouse IgG: https://www.scbt.com/p/normal-mouse-igg?productCanUrl=normal-mouse-igg&_requestid=1521791<br>-Rabbit IgG, polyclonal-Isotype Control: https://www.abcam.com/rabbit-igg-polyclonal-isotype-control-ab37415.html<br>-Goat anti-Rabbit Light Chain Secondary Antibody: https://www.novusbio.com/products/light-chain-antibody_nbp2-75935<br>-Goat Anti-Mouse IgG, Light-Chain Specific Antibody: https://www.cellsignal.com/products/secondary-antibodies/goat-anti-mouse-igg-light-chain-specific-antibody-hrp-conjugate/91196?site-search-type=Products&N=4294956287&Ntt=91196s&fromPage=plp&_requestid=1751660<br>-Anti-PRDM16 antibody: https://www.rndsystems.com/cn/products/human-mouse-prdm16-mel1-antibody_af6295

Eukaryotic cell lines

Policy information about cell lines

Cell line source(s)	Stromal vascular fraction (SVF) from the inguinal WAT of C57BL/6J mice, Prdm16 flox/flox mice, and Appbp2flox/flox mice were immortalized by expressing the SV40 Large T antigen.<br>SF9 cells were obtained from UC Berkeley Cell Culture Facility.

	HEK293T, HEK293 virus packaging cells, C2C12 were purchased from ATCC.
Authentication	RNA-sequencing of the cell lines provide authentification.
Mycoplasma contamination	All the cell lines were routinely tested for mycoplasma infection and confirmed as negative for mycoplasma contamination.
Commonly misidentified lines (See ICLAC register)	No commonly misidentified cell line was used.

Animals and other organisms

Policy information about studies involving animals; ARRIVE guidelines recommended for reporting animal research

Laboratory animals	Adult mice aged 6-74 weeks were used for all the experiments. Littermate controls with same sex were used. All the mice had free access to food and water, and were housed under 12-hour light/dark cycle, at 22 ℃, and 45% humidity in average. Cul2flox/- mice in the C57BL/6J background were generated by Applied StemCell using CRISPR-Cas9 technology. Appbp2 flox/- mice in the C57BL/6J background were made by Cyagen company with CRISPR-Cas9 technology. All the mice used in the study were C57Bl6J background. For the metabolic studies, male mice at 6 weeks old were on a 60% high-fat diet for up to 14 weeks under a room temperature. Appbp2 knock-in mice that carry the S561N mutation (equivalent human SNP found in the APPBP2 gene) were generated by Cyagen Inc. To recapitulate the human S561N variant of APPBP2 in mice (see Extended Fig. 10a), we mutated S561 to N and S562 to T by co-injecting the gRNA and the donor oligo containing p.Y551 (TAT to TAC) and p.A556 (GCC to GCG) into fertilized mouse eggs. Adipocyte-specific Cul2 KO (Adipo-Cul2 KO mice) or Appbp2 KO mice (Adipo-Appbp2 KO mice) were developed by crossing Cul2 or Appbp2 floxed mice with Adiponectin-Cre mice (B6; FVB-Tg (Adipoq-Cre)1Evdr/J, stock 028020).
Wild animals	This study did not involve wild animals
Field-collected samples	No field collected samples.
Ethics oversight	All the animal experiments in this study were performed in compliance with protocols approved by the Institutional Animal Care and Use Committee (IACUC) at UCSF and Beth Israel Deaconess Medical Center.

Note that full information on the approval of the study protocol must also be provided in the manuscript.
