# Supplementary material for: Post-translational control of beige fat biogenesis by PRDM16 stabilization
Source: Nature. 2022 Aug 17;609(7925):151–8. doi: 10.1038/s41586-022-05067-4 (PMC9433319; doi:10.1038/s41586-022-05067-4)
Supplement: Supplementary file 3 — Sequences of primers used for mouse genotyping, gRNA, shRNA, RT–qPCR and ChIP–qPCR. [file 41586_2022_5067_MOESM3_ESM.pdf]

Supplementary Table 1

Sequences of genotyping primers

AdipoQ-Cre alleles	ACGACCAAGTGACAGCAATG	CTCGACCAGTTTAGTTACCC
Cul2 alleles	CTGCAACCCTGTTCTTTTTATTCAATTAAAGTAT	GCTGTGTTGCTTCTGTCTTTTATGTG
Appbp2 alleles-5′ flox	AAAAGTGTACAGCAGCCATCTCTT	AAGCGAAGCCTATACTTCAGAAAG
Appbp2 alleles-3′ flox	CTTCCCTTGTTTGACCATTTAGT	AGAGAAAGTGTGGCTATCATCAT
Appbp2 knockin	TGTGTTGCTTTAGGGAAGAAACTG	TTCTACAGGAACTGCAATACAGGA

Sequences of shRNAs

Scrambled control	CAACAAGATGAAGAGCACCAA
sh-*Appbp2* #1	GAATATCCAGTTCGACGTTTA
sh-*Appbp2* #2	GCTCTATCAGTCAATGAGAAA
sh-*Zyg11* b	GGTAACCAGATGCGCCTAAAG
sh-*Klhdc3*	CCTGGGCAAGATCATGTACAT
sh-*Klhdc10*	CGGCTACATCTATAGCACAGA
sh-*Lrr1*	CCTTCAAGAGCTTAACCTTAA

gRNA target sequence for generation of $Cul2^{flox/-}$ mice

gRNA1 (matching forward strand of gene)	GTCTTGCCAGTGCACTAGGAAGG
gRNA2 (matching forward strand of gene)	CCTAGATCACTGATTTACAGTGG

gRNA target sequence for generation of $Appbp2^{flox/-}$ mice

gRNA1 (matching forward strand of gene)	GAAGGCTTCAGGATACACCTGGG
gRNA2 (matching forward strand of gene)	TGATAGCAAATGCCATGGCGTGG

gRNA target sequence for generation of Appbp2 knockin mcie

gRNA (matching reverse strand of gene)	GGCTGCTGCTGACGTCTTCAAGG

Sequences of real-time qPCR and ChIP-qPCR primers

	Gene	Forward Primer	Reverse Primer
	Tbp	ACCCTTCACCAATGACTCCTATG	TGACTGCAGCAAATCGCTTGG
	36B4	GGCCCTGCACTCTCGCTTTC	TGCCAGGACGCGCTTGT
	Cul2 for shRNA knockdown	ACCGCTTCTCAGATATCTAT	TTCTTCCCAGTACCTATGAT
	Cul2 for floxed mice knockout	ACCGCTTCTCAGATATCTAT	GAACTCTCTTATACAAATGC
	Appbp2 for shRNA knockdown	GTTGTGTGAGGTTGCTTCAT	CTATTTGCTTGCTGGCCATG
	Appbp2 for floxed mice knockout	GACGTTTACTACAAGCTTTA	GGAGCAAATGTCTTTTATCC
	Prdm16	CAGCACGGTGAAGCCATTC	GCGTGCATCCGCTTGTG
	Zyg11b	GCATTCCACGGTCTCCTGAA	AAGTTCTACTAGCTTGTGGT
	Klhdc2	GGCTACAAGAGTAATCAAGT	GGGAACGTCACCTTCAGTGT
	Klhdc3	CAATGCTGTGTCCTTGCGTT	ACGGTGTCATCAATGAGGAC
	Klhdc10	TTCTTGAGAGGCCACAGACC	TTCATCATAGTCTGGGTTAT
	Lrr1	CAGCTACAAGCTAAGAGAGA	CAGGATTGGCCTTACTCAGA
	Lrrc14	AACTGCTCAGCACCCTGCAG	CCAACTTTTTGAGATGGGCA
	PGC1a	AGCCGTGACCACTGACAACGA	GCTGCATGGTTCTGAGTGCT
	Ucp1	CACCTTCCCGCTGGACACT	CCCTAGGACACCTTTATACCTAATGG
	Cidea	ATCACAACTGGCCTGGTTACG	TACTACCCGGTGTCCATTTCT
	Cox8b	GAACCATGAAGCCAACGACT	GCGAAGTTCACAGTGGTTCC
	Cox7a	CAGCGTCATGGTCAGTCTGT	AGAAAACCGTGTGGCAGAGA
	AdipoQ	GCACTGGCAAGTTCTACTGCAA	GTAGGTGAAGAGAACGGCCTTGT

Mouse	*Fabp4*	ACACCGAGATTTCCTTCAAACTG	CCATCTAGGGTTATGATGCTCTTCA
	Kcnk3	ACGGAGGCAAGGTGTTCTG	ACGACACGAAACCGATGAGC
	Pparg	TGAAAGAAGCGGTGAACCACTG	TGGCATCTCTGTGTCAACCATG
	Dio2	CAGTGTGGTGCACGTCTCCAATC	TGAACCAAAGTTGACCACCAG
	Elovl3	GATGGTTCTGGGCACCATCTT	CGTTGTTGTGTGGCATCCTT
	F4/80	TTTCCTCGCCTGCTTCTTC	CCCCGTCTCTGTATTCAACC
	CD11c	AAAATCTCCAACCCATGCTG	CACCACCAGGGTCTTCAAGT
	CD206	CAAGGAAGGTTGGCATTTGT	CCTTTCAGTCCTTTGCAAGC
	TGF beta1	CTCCCGTGGCTTCTAGTGC	GCCTTAGTTTGGACAGGATCTG
	Mmp2	GGACAAGTGGTCCGCGTAAA	CCGACCGTTGAACAGGAAGG
	Mmp3	ACATGGAGACTTTGTCCCTTTTG	TTGGCTGAGTGGTAGAGTCCC
	Mmp12	CTGCTCCCATGAATGACAGTG	AGTTGCTTCTAGCCCAAAGAAC
	Tnfa	ACACTCAGATCATCTTCTCAAAATTCG	GTGTGGGTGAGGAGCACGTAGT
	Ccl2	AGGTCCCTGTCATGCTTCTG	GCTGCTGGTGATCCTCTTGT
	Lox	CAGCCACATAGATCGCATGGT	GCCGTATCCAGGTCGGTTC
	Col3a1	CTGTAACATGGAAACTGGGGAAA	CCATAGCTGAACTGAAAACCACC
	Pcolce2	TGTGGCGGCATTCTTACCG	CCCTCAGGAACTGTGATTTTCCA
	Prdm16	CAGCACGGTGAAGCCATTC	GCGTGCATCCGCTTGTG
	Acyl	GTGGCCCCAACTATCAAAGAG	ATGGGATCCCAGTGGTC
	Acaca	AGCAACATCACATCAGTCCTGT	CAGTGTAGCTGCATGACTATCTAGG
	Srebp1c	TCTGCCTTGATGAAGTGTGG	AGCAGCCCCTAGAACAAACA
	Acc	AATGAACGTGCAATCCCATTTG	ACTCCACATTTGCGTAATTGTTG
	Fasn	GAGGTGGTGATAGCCGGTAT	TGGGTAATCCATAGAGCCCAG
	Scd1	AGGCCTGTACGGGATCATACT	AGAGCGCTGGTCATGTAGTAG
	Cox1	TAGCCCATGCAGGAGCATCA	TGGCTGGGGGTTTCATGTTGA
	Cox2	ACCTGGTGAACTACGACTGCT	CCTAGGGAGGGGACTGCTCA
	Cox3	CTTCACCATCCTCCAAGCTTCA	AGTCCATGGAATCCAGTAGCCAT
	Atp6	TGGCATTAGCAGTCCGGCTT	ATGGTAGCTGTTGGTGGGCT
	Atp8	TTCCCACTGGCACCTTCACC	TGTTGGGGTAATGAATGAGGCAA
	Ins1	GGACCCACAAGTGGAACAAC	GTGCAGCACTGATCCACAAT
	Cidea (13)	GGCCACTTGAGGAGCCAACCA	TGGGCACTGGCCTTGTAGCTG
	Ppara (11)	AAGAGCATGGGACAGTGGCCG	TGGCCAGCTGAAGGTCACCAC
	Ppara (14)	CCTGCCCCATAGGCAGTATGGTC	ACAGGGGCAGAAGCCAAGCTG
	Ppargc1a (38)	TCCGAGTTTCCCTGCTGTGGC	AGGGACTTGCAGCTGTGGTGG
	Ppargc1a (42)	GAGGTGGCACCAGGACACCAG	CCCAAGCTCGAGACTCCGCTC
	Ucp1 (2.5)	CAAATGGTGACCGGGTGCCCT	GGGTGACTGACCCTCTGTGACG
	Ucp1 (4.7)	CCCCACTGCCTGTCACGTTCA	GAAGCTGCCGAATGGTGCGTC
	Ucp1 (5.7)	ACCACACCATTTGGAGCCTGAC	TGAGTTTGCAGGGAGGATGGGC
Human	*PRDM16*	ATCCACAACAAGGAGAAGCC	TGCTGGCTCACTGGTGCGTT
